# A Reference List of Phenolic Compounds (Including Stilbenes) in Grapevine (*Vitis vinifera* L.) Roots, Woods, Canes, Stems, and Leaves

**DOI:** 10.3390/antiox9050398

**Published:** 2020-05-08

**Authors:** Piebiep Goufo, Rupesh Kumar Singh, Isabel Cortez

**Affiliations:** 1 Centre for the Research and Technology of Agro-Environment and Biological Sciences, Departamento de Agronomia, Universidade de Trás-os-Montes e Alto Douro, Quinta de Prados, 5000-801 Vila Real, Portugal; icortez@utad.pt; 2 Centro de Química de Vila Real, Universidade de Trás-os-Montes e Alto Douro, Quinta de Prados, 5000-801 Vila Real, Portugal; rupesh@utad.pt

**Keywords:** bioactive compounds, vegetative organs, antioxidant activity, *Vitis vinifera*, secondary metabolites, polyphenol database, grapevine

## Abstract

Due to their biological activities, both in plants and in humans, there is a great interest in finding natural sources of phenolic compounds or ways to artificially manipulate their levels. During the last decade, a significant amount of these compounds has been reported in the vegetative organs of the vine plant. In the roots, woods, canes, stems, and leaves, at least 183 phenolic compounds have been identified, including 78 stilbenes (23 monomers, 30 dimers, 8 trimers, 16 tetramers, and 1 hexamer), 15 hydroxycinnamic acids, 9 hydroxybenzoic acids, 17 flavan-3-ols (of which 9 are proanthocyanidins), 14 anthocyanins, 8 flavanones, 35 flavonols, 2 flavones, and 5 coumarins. There is great variability in the distribution of these chemicals along the vine plant, with leaves and stems/canes having flavonols (83.43% of total phenolic levels) and flavan-3-ols (61.63%) as their main compounds, respectively. In light of the pattern described from the same organs, quercetin-3-*O*-glucuronide, quercetin-3-*O*-galactoside, quercetin-3-*O*-glucoside, and caftaric acid are the main flavonols and hydroxycinnamic acids in the leaves; the most commonly represented flavan-3-ols and flavonols in the stems and canes are catechin, epicatechin, procyanidin B1, and quercetin-3-*O*-galactoside. The main stilbenes (*trans*-ε-viniferin, *trans*-resveratrol, isohopeaphenol/hopeaphenol, vitisin B, and ampelopsins) accumulate primarily in the woods, followed by the roots, the canes, and the stems, whereas the leaves, which are more exposed to environmental stresses, have a low concentration of these compounds. Data provided in this review could be used as (i) a metabolomic tool for screening in targeted and untargeted analyses and (ii) a reference list in studies aimed at finding ways to induce naturally occurring polyphenols on an industrial scale for pant and human disease control.

## 1. Introduction

Grapevine (*Vitis vinifera* L.) is a perennial woody fruit crop used for wine, juice, fresh consumption (table grapes), dried fruit, and distilled liquor [1,2,3,4,5,6,7,8]. Most of the premium cultivars are highly susceptible to several pathogenic microorganisms [6,9,10,11,12,13]. In the past decades, the understanding of grapevine/pathogen interactions has focused on the molecular response of the host, and several metabolites, proteins, and gene/gene products have been identified as putative biomarkers of grapevine disease tolerance [14,15,16,17,18]. In particular, the importance of phenolic compounds as natural fungicides implicated in the resistance of some grapevine cultivars to fungi, oomycetes, bacteria, phytoplasma, and viruses have been highlighted by several authors; one of the most known properties of these compounds is their antioxidative activity, whereby they are able to scavenge free radicals and positively influence health outcomes [5,19,20,21,22,23,24,25,26,27,28,29,30,31]. Plants have evolved a variety of mechanisms using phenolic compounds, including the formation of a protective shield against ultraviolet (UV) radiation. Therefore, the compounds produced by highly resistant varieties are of great interest for the development of improved crops, natural spray reagents, and new dietary supplements or pharmaceuticals [5,19,32,33,34,35,36].

In *V. vinifera*, many studies have been published that reported on the concentration of phenolics in berry-containing foods and their impact on human health [18,35]. Indeed, several databases are available for the levels of phenolic compounds in the fruits (e.g., eBASIS, Phenol-Explorer), but none are available for non-edible parts of *V. vinifera*. Recently, the stems and canes of this economically important genus have been reported as an untapped source of health-promoting compounds [16,17,31,35,36,37,38,39]. Because of this, numerous efforts for isolation, identification, and quantification of phenolic compounds in the vegetative organs of grapevine have been ongoing. In order to properly design valorization strategies, the precise chemical composition of these vegetative materials has to be known. In this review, a more thorough understanding of the chemical diversity of polyphenols within *V. vinifera* vegetative organs is provided, which will be useful in this endeavor. The review includes an overview of compounds identified in the roots, cordon and trunk woods, canes, stems, and leaves with their mass and UV spectrum patterns, followed by an estimation of their levels. It concludes with a brief presentation of factors affecting the biosynthesis and accumulation of these compounds. The fallout of such data is multifaceted and will surely contribute to advancing the scientific knowledge in the field.

## 2. The Vegetative Organs of the Vine Plant

Grapevine is a climber whose growth in the vineyard is maintained with pruning in order to control the quantity and quality of the grapes [40]. Like any other plant, grapevine has vegetative and reproductive organs. The vegetative organs of vine include the roots and five parts extending from the root system and visible aboveground: trunk, cordons, canes, stems, and leaves. These organs play a key role in light energy capture via photosynthesis, as well as water and nutrient absorption as regulated by transportation.

### 2.1. Roots

The roots of a vine plant are multi-branched structures that grow to various depths into the soil on the basis of the variety (rootstock), and are responsible for anchoring the plant to the ground [12,23,29,30,41,42].

### 2.2. Woods

In the literature, the “wood” refers to samples obtained from the trunk and the cordons. The trunk is composed of sleeves of conductive tissues, most notably the phloem and the xylem [13,43,44,45]. Cordons or “arms” are extensions of the trunk and the parts where canes (one-year-old wood containing between 8 and 15 buds) and spurs (one-year-old wood containing between two and three buds) originate [12].

### 2.3. Canes

The terms “stems”, “canes”, “stalks”, and “shoots” are sometimes used interchangeably in the literature. For the purpose of this review and on the basis of the literature surveyed, the shoot is the new green growth that develops from buds located on the cordons [24,25,46,47,48,49]. Once the leaves fall from the vine at the beginning of the dormant season, the brown and harden/woody shoot is considered a cane, which represents a large source of waste derived from the wine industry [40,50,51,52].

### 2.4. Stems

The stem consists of the stalk extending out to hold the grape cluster (also known as the bunchstem) and the “stem” of the individual grape berry (also called the pedicel by some authors) [9,37,50,53,54,55,56,57,58].

### 2.5. Leaves

Leaves are the most visible parts of the canopy and consist of the blade (the broad, flat part of the leaf designed to absorb sunlight and CO_2_), and the petiole (the stem-like structure that connects the leaf to the shoot) [4,5,11,19,22,32,59,60,61,62,63,64].

## 3. Extraction, Separation, and Identification of Phenolic Compounds in Grapevine

In grapevine varieties, polyphenols are present as constitutive compounds of the lignified organs (roots, canes, seeds, stems, ripe cluster stems) and/or as induced substances in leaves and berries. In the frame of a long-term project aimed at investigating the physiological and molecular responses of grapevine to trunk diseases [15], several papers that contained the terms “grapevine, grape, vine, vineyard, or vitis” in their titles, plus one of the following terms: “phenolic, polyphenol, flavonoid, anthocyanin, proanthocyanidin, tannin, stilbene, stilbenoid, bioactive, bioactivity, antioxidant, antioxidative, metabolite, metabolic, metabolomic, metabolome, leaf, stem, root, wood, cordon, cane, trunk, phytoalexin, defense, resistance”, or terms related to the specific diseases and pathogens of grapevine, were retrieved from citation databases; 80 papers were analyzed that primarily reported on the presence and levels of polyphenols in the vegetative organs (Tables S1 and S2). The term “polyphenol” is used in this review to indicate both the compounds with a second aromatic ring and those arising from the polymerization of flavonoidic/catechin units. Despite their structural diversity, all polyphenols share a common structure element, which consists of a benzene ring to which more than one hydroxyl group is attached [65].

The surveyed literature shows that many extraction methods have been tested, and that several analytical methods using numerous techniques have been developed for the investigation of polyphenols in grapevine, including high-performance liquid chromatography (HPLC) coupled with diode array detection (LC–DAD), HPLC coupled with mass spectrometry (LC–MS, LC–MS/MS), and nuclear magnetic resonance (NMR) [24,28,36,66,67]. Different advantages and disadvantages are associated with each analytical system. Analysis by HPLC–DAD (or HPLC/UV–VIS) is limited by similar or identical absorption maxima of target compounds belonging to the same structural class of polyphenols. Other problems such as lack of baseline resolution, leading to overestimation of individual compound levels, may exist, along with poor sensitivity [16,17,36]. Because of its high selectivity, LC–MS/MS with electrospray ionization (ESI), atmospheric pressure photoionization (APPI), or chemical ionization (APCI) enables the sensitive and simultaneous detection and identification of a large number of (even co-eluting) compounds from a single chromatogram and is therefore the method of choice when libraries are available. MS also enables reductions in the process of sample preparation from extracts [10,68]. NMR, on the other hand, is a non-destructive high throughput method that allows metabolite identification and quantification. It is, however, significantly less sensitive than MS, although more reproducible, especially in long-term studies where samples collected and analyzed over different time periods have to be compared. NMR is also an invaluable tool for the de novo structure determination of compounds [16,59]. In all cases, however, precise conditions are required to achieve a complete qualitative survey of all metabolites over a significant dynamic range in a complex plant extract. Depending on the optimization of extraction and detection parameter settings, two large groups of chemical compounds with phenolic characteristics—that are classified into several structure classes—are clearly delineated in grapevine and are separately discussed in this paper.

The first group comprises phenolic acids (hydroxybenzoic and hydroxycinnamic acids), flavonoids (e.g., flavonols, anthocyanins, proanthocyanidins), and coumarins, which are usually present as preformed compounds in the tissues. Indeed, HPLC in gradient mode on reversed phase C18 columns provides a means to separate most of these compounds in a single chromatography run without the need for derivatization. Due to their structural complexity, however, proanthocyanidins are more easily separated alone by hydrophilic interaction liquid chromatography (HILIC) according their degree of polymerization, or by reverse-phase chromatography, although some of them coelute [69].

The second group is constituted of stilbenic compounds (stilbenes that bear the core structure of 1,2-diphenylethylene and stilbenoids that are hydroxylated derivatives of stilbenes). Several of these compounds are produced naturally by several plants upon attack by pathogens [8,14,70,71]. Because of their dynamic behavior as responses to stresses, the detection of stilbenes requires methods that can be used for monitoring their differential response in various phytopathologic situations [61,71]. Their extraction generally requires specialized instrumentation and expertise, for instance, sample cleaning techniques such as solid phase extraction (SPE), sample dilution, selective extraction, or use of stable isotopes. Most of the qualitative or quantitative analytical studies of stilbenes are performed with HPLC and in an increasing sensitivity order UV, fluorescence (FD), electrochemical (ECD), or MS detection [36,50,52]. A method for the simultaneous separation of proanthocyanidins and stilbenoids has been reported, using a comprehensive bi-dimensional chromatography, with a diol stationary phase in the first dimension and a C18 stationary phase in the second dimension [50,72].

## 4. Polyphenols (Excluding Stilbenes) Identified in the Vegetative Organs of Grapevine

Phenolic compounds produced by grapevine range from cell wall-thickening compounds such as lignin and tannins, to specialized compounds such as phenolic acids and flavonoids. The chemical characterization of these compounds is based on analysis of different groups of components individually by LC–MS, mainly in negative ionization mode, although some LC–MS methods in positive-ion mode have been reported [16]. With high-resolution MS, compounds are identified by processing raw data with specific algorithms to calculate molecular formulae on the basis of the monoisotopic mass of the [M–H]^−^ ion and the relative abundances and distances (spacing) of m/z signals measured in the isotopic pattern. Metabolites are then identified by searching in the available MS databases, in comparison with UV spectra patterns reported in the literature [16]. In Table 1, a database specific to grapevine phenolics containing 105 metabolites, including their specific MS and UV information, is provided.

### 4.1. Hydroxycinnamic Acids

The phenylpropanoid pathway starts with the aromatic amino acid phenylalanine and leads to derivatives with one, two, or more aromatic rings (C6), each ring with a characteristic substitution pattern, and with different modifications of the propane residue of phenylalanine (C3) [35]. At least 15 hydroxycinnamic acids (moiety C6–C3) have been identified in the vegetative organs of grapevine, with different degrees of hydroxylation and methylation of C6. These include caftaric, coutaric, chlorogenic, chicoric, fertaric, caffeic, *p*-coumaric, ferulic, sinapic, and cinnamic acids, and some of their derivatives, that is, 1-*O*-sinapoyl-*β*-D-glucose, 1-*O*-(4-coumaroyl)-glucose, 1-caffeoyl-*β*-D-glucose (reported as caffeic acid derivative by some authors), ferulic acid pentose (reported as ferulic acid derivative by some authors), and a caftaric acid isomer (Table 1) [20,32,46,47,53,73,74].

### 4.2. Hydroxybenzoic Acids

The cleavage of a C2 fragment from the aliphatic side chain of *p*-coumaric acid leads to hydroxybenzoic acids (C6–C1) [35], and nine have been reported in the vegetative organs of grapevine: quinic, gallic, protocatechuic, *p*-hydroxybenzoic, gentisic, γ-resorcylic, vanillic, syringic, and ellagic acids, mostly detected in the leaves (Table 1) [4,32,47,73,75].

### 4.3. Flavan-3-Ols or Flavanols

The condensation of three C2 residues with an activated hydroxycinnamic acid produces metabolites with a second aromatic ring linked to the phenylpropanoid moiety, with a common C6-C3-C6 skeleton of flavonoids. The basic flavonoid chemical structure is the flavan nucleus, consisting of 15 carbon atoms arranged in two benzene rings (A and B) linked via a heterocyclic oxygen-containing pyran ring (C). The main classes of flavonoids differ in the level of oxidation and saturation of the C ring, the most relevant being flavan-3-ols including proanthocyanidins, anthocyanins, flavanones, flavonols, and flavones [35,65]. Flavan-3-ols exhibit a saturated C-ring hydroxylated in the 3-position. The A-ring of flavan-3-ols is generally hydroxylated in C5 and C7 and the B-ring in C4. Diversity arises from the substitution pattern of the B-ring and can be increased by galloylation and glucosylation of the 3-hydroxyl group [76]. The presence of two asymmetric carbons (in C2 and C3) opens the possibility for different stereoisomers, that is, 2*R*,3*S* (2,3-*trans*), 2*R*,3*R* (2,3-*cis*), 2*S*,3*R* (2,3-*trans*), and 2*S*,3*S* (2,3-*cis*) configurations. The following eight flavanol monomers are reported in grapevine leaves, stems, and canes: catechin, gallocatechin, epigallocatechin, epigallocatechin gallate, epicatechin, gallocatechin gallate, epicatechin gallate, and catechin gallate (Table 1) [32,59,69,73,77].

### 4.4. Proanthocyanidins

Proanthocyanidins, also known as condensed tannins, are both oligomeric and polymeric compounds arising from flavanol condensation. Linkages between constitutive flavan-3-ol units are found between C4 and C6 or C4 and C8 in the case of B-type proanthocyanidins. A-type are linked with additional C2-*O*-C7 or C2-*O*-C5 bonds. Substitution in the 4-position gives rise to another asymmetric center on extension and upper units, but the usual configuration is 3,4-*trans* (i.e., 3*S*,4*S* or 3*R*,4*S*). The chain length of one polymer is described by the degree of polymerization (DP), and the mean degree of polymerization (mDP) of a heterogeneous population of polymers [76]. The following nine proanthocyanidins are reported in grapevine leaves, stems, and canes: procyanidin A1, procyanidin B1, procyanidin B2, procyanidin B3, procyanidin B4, procyanidin C1, procyanidin T2, prodelphinidin A-type (reported as epigallocatechin-epicatechin dimer by some authors), and a procyanidin dimer gallate (Table 1) [33,50,53,69,76,77].

### 4.5. Anthocyanins

Anthocyanins share the same molecular structure of flavonoids composed by one heterocyclic benzopyran ring (as the C ring), one fused aromatic ring (as the A ring), and one phenyl constituent (as the B ring). Nevertheless, they differ on the basis of hydroxyl or methoxyl substitutions in the lateral phenyl B ring, and, in general, for glycosylations and esterifications. Anthocyanins of *Vitis* are structurally based on five aglycones/anthocyanidins—malvidin, cyanidin, delphinidin, peonidin, and petunidin—which differentiate on the basis of number and position of their hydroxyl groups and their degree of methylation. Acylation occurs at the C6 position of the glucose molecule by esterification with acetic, *p*-coumaric, and caffeic acids [77,78]. Anthocyanins have been mainly reported in the leaves of grapevine (at least 14) and include: delphinidin-3-*O*-glucoside, cyanidin-3-*O*-glucoside, cyanidin-3-(6-*O*-coumaroyl)glucoside, petunidin-3-*O*-glucoside, petunidin-3-(6-*O*-acetyl)glucoside, petunidin-3-(6-*O*-coumaroyl)glucoside, peonidin-3-*O*-glucoside, peonidin-3-(6-*O*-acetyl)glucoside, peonidin-3-(6-*O*-coumaroyl)glucoside, malvidin-3-*O*-glucoside, malvidin-3-(6-*O*-acetyl)glucoside, malvidin-3-(6-*O*-coumaroyl)glucoside, malvidin-3-(6-*O*-caffeoyl)glucoside, and malvidin-3-*O*-rutinoside (Table 1) [4,5,77,78,79].

### 4.6. Flavones

Flavones are the simplest members of the class of flavonoids and consist of 4H-chromen-4-one bearing a phenyl substituent at position 2 [65]. Among the flavonoids naturally occurring in grapevine, flavones represent the least common group of aromatic compounds with only apigenin-7-*O*-glucoside and luteolin-7-*O*-glucoside reported in the leaves (Table 1) [4].

### 4.7. Flavonols

Chemically, flavonols or 3-hydroxyflavones differ from many other flavonoids in that they have a double bond between positions 2 and 3 and an oxygen (a ketone group) in position 4 of the C ring, like flavones; however, they differ from flavones due to the presence of a hydroxyl group at the position 3. Most of the flavonols exist as *O*-glycosides and seldomly as *C*-glycosides, and their conjugated derivatives (glycones) are mainly bound to sugars, hydroxycinnamic acids, or organic acids [35]. Flavonols make up the largest group of flavonoid compounds encountered in grapevine leaves and stems, with at least 35 compounds reported in the literature (Table 1) [1,2,4,5,32,34,59,66,77,79,80] derived from four aglycones: myricetin, quercetin, kaempferol, and isorhamnetin:

- Myricetin, myricetin-3-*O*-galactoside, myricetin-3-*O*-glucuronide, myricetin-3-*O*-glucoside, and myricetin-3-*O*-rhamnoside;
- Quercetin, quercetin-3-*O*-rutinoside, quercetin-3-*O*-galactoside, quercetin-3-*O*-glucoside, quercetin-3-*O*-glucuronide, quercetin-3-*O*-rhamnoside, quercetin-3-(6-*O*-acetyl)glucoside, quercetin-3-(3-*O*-arabinosyl)glucoside, quercetin-3-(7-O-glucosyl)glucuronide, quercetin-3-*O*-arabinose (reported as quercetin-*O*-pentoside by some authors), quercetin-3-(3-*O*-rhamnosyl)glucoside-7-*O*-rhamnoside, quercetin-3-(6-*O*-rhamnosyl)galactoside, and diquercetin-3-(3-*O*-glucosyl)glucuronide;
- Kaempferol, kaempferol-3-*O*-galactoside, kaempferol-3-*O*-rutinoside, kaempferol-3-*O*-glucuronide, kaempferol-3-*O*-glucoside, kaempferol-3-*O*-xyloside (or kaempferol-*O*-pentoside by some authors), kaempferol-3-*O*-rhamnoside, dihydrokaempferol-3-*O*-rhamnoside, kaempferol-3-(6-*O*-coumaroyl)glucoside, and kaempferol-3-(7-*O*-glucosyl)galactoside (or kaempferol-3,7-diglucoside by some authors);
- Isorhamnetin-3-*O*-galactoside, isorhamnetin-3-*O*-glucoside, isorhamnetin-3-*O*-arabinose (or isorhamnetin-*O*-pentoside by some authors), isorhamnetin-3-*O*-glucuronide, isorhamnetin-3-*O*-rutinoside, isorhamnetin-3-(6-*O*-feruloyl)glucoside, and isorhamnetin-3-(4-*O*-rhamnosyl)rutinoside (or isorhamnetin diglycoside by some authors).

### 4.8. Flavanones

Flavanones (also called 2,3-dihydroxyflavones) lack the double bond between carbons 2 and 3 in the C-ring of the flavonoid skeleton, which is present in flavones and flavonols. Thus, flavanones are chiral at the C2 position, and are generally glycosylated by glucoside or disaccharide at position seven to give flavanone glycosides [65]. The following eight flavanones have been reported in the vine plant: taxifolin, taxifolin-*O*-pentoside, taxifolin-3-*O*-glucoside, taxifolin-3-*O*-rhamnoside, hesperetin, eriodictyol-7-*O*-glucoside, naringenin, and naringenin-7-*O*-glucoside (Table 1) [4,74].

### 4.9. Coumarins and Dihydrochalcones

Coumarins are 1,2-benzopyrones (fused benzene and α-pyrone rings) that are derived from the phenylpropanoid pathway, but can also be produced through the cleavage of *O*-hydroxycinnamic acid that exist in free or glycosylated forms. In studies aimed at identifying polyphenols in grapevine, the following compounds have been detected: aesculin, fraxin, aesculetin, umbelliferone (coumarins), and phlorizin (dihydrochalcone) (Table 1) [19,32,81,82].

### 4.10. Non-Phenolic Compounds

The literature surveyed reveals that at least eight non-phenolic compounds or volatile compounds are usually eluted with phenolic compounds, and these include pyrogallol and catechol (benzenediols), sinapaldehyde, syringaldehyde and coniferaldehyde (hydroxycinnamaldehydes), vanillin and acetovanillone (benzaldehydes), and arbutin (hydroquinone) (Table S1) [35,46,47,55,60,83]. Moreover, some still unknown compounds with phenolic characteristics have been reported, and their importance can be estimated only if their chemical structure is determined.

## 5. Stilbenic Compounds Identified in the Vegetative Organs of Grapevine

The condensation of three C2 residues with an activated hydroxycinnamic acid (as with flavonoids) produces stilbenes, which are metabolites with an essential structural skeleton of two aromatic rings joined by an ethylene bridge (C6–C2–C6) [31,50]. Stilbenes emit a blue fluorescence under UV light with excitation and emission peaks around 320 and 390 nm, respectively [52,63]; in fact, the name “stilbene” derives from the Greek word “*stilbos*”, which is translated as “shining” [16,36]. The chemical structure of stilbenes in both the monomeric and oligomeric states is constituted by a diphenylethylene group oriented in *trans* or *cis*. The presence of a *cis*-stilbenic chromophore gives rise to different spectra, with an absorption maximum of lower intensity and of shorter wavelength compared with that of the *trans*-isomer [61]. Light exposition of *trans-*stilbene solutions has been shown to partially photoisomerize stilbenes into *cis* forms [7,59,61]. There are several areas of confusion with stilbene nomenclature. According to current practice however, the *trans*/*cis* nomenclature is used to describe the stereochemistry at saturated rings, whereas the Z/E nomenclature is used to describe the stereochemistry of double bonds [8,13,61,64]. In this review, the *trans*/*cis* nomenclature is used, although at least two compounds have been reported with other nomenclatures, namely, miyabenol C and ε-viniferin. Both *trans*-E-miyabenol C and *trans*-Z-miyabenol C are reported in the literature [61]. In the case of ε-viniferin, there are two stereochemical centers, at positions 7a and 8a on the dihydrofuran ring, allowing for four potential stereoisomers: (+)-*trans*-ε-viniferin, (-)-*trans*-ε-viniferin), (+)-*cis*-ε-viniferin, and (-)-*cis*-ε-viniferin) [8].

Most stilbenes have been identified using NMR and MS, which are the most informative techniques. Using MS, the initial identification of compounds is performed on the exact mass measurement of the monoisotopic ion and isotopic pattern, enabling the molecular formula to be identified with a high-confidence score and low mass error. Exact mass measurements of MS/MS fragments either confirm or deny the putative structure. Because MS/MS cannot distinguish between isomeric compounds, tentative assignment is also based on comparisons with data found in the literature [13,16] and NMR profiles. In addition, UV−VIS data based on λmax and UV spectrum when available are compared with those in the literature [12,61]. With this approach, a total of 78 stilbenes have been successfully identified in the vegetative tissues of the vine plant. A database of these stilbenes is provided in Table 2; the masses, when available, are those derived from the negative ion LC−MS datasets. Mass data are usually in agreement among publications, with minor changes in product ions owing to different fragmentation conditions.

The basic simple structure of stilbenes gives rise to a wide array of compounds that primarily vary in the number and position of hydroxyl groups and various substitutions with sugars, methyl, and methoxy groups, in addition to the structural conformations of the molecules and oligomerization patterns [8,31,39,68].

### 5.1. Monomeric Stilbenes

Of the total known *V. vinifera* stilbenes, 23 are monomers: *trans*-astringin, *cis*-astringin, *trans*-resveratroloside, *cis*-resveratroloside, *trans*-resveratrol-2-*C*-glucoside, *trans*-resveratrol-10-*C*-glucoside, *trans*-resveratrol-*O*-glucoside, *cis*-resveratrol-*O*-glucoside, *trans*-piceid, *cis*-piceid, *trans*-piceatannol, *trans*-isorhapontin, *trans*-resveratrol, *cis*-resveratrol, 2,4,6-trihydroxyphenanthrene-2-*O*-glucoside, *trans*-isorhapontigenin, *cis*-isorhapontigenin, *trans*-pinostilbene, *cis*-pinostilbene, *trans*-pinostilbene-4′-*O*-glucoside (or *trans*-pinostilbene-3-*O*-glucoside by some authors), *trans*-pterostilbene, *cis*-pterostilbene, and *trans*-rhaponticin (or *trans*-rhapontin by some authors) (Table 2) [7,31,40,50,53].

### 5.2. Dimeric Stilbenes

The majority of the stilbenoids in grapevine vegetative organs are dimers (30 in total): leachianol G, leachianol F, restrytisol A, ampelopsin A, ampelopsin D, ampelopsin F, pallidol, caraphenol B, quadrangularin, (+)-*trans*-ε-viniferin (and occasionally (-)-*trans*-ε-viniferin), (+)-*cis*-ε-viniferin (and occasionally (-)-*cis*-ε-viniferin), viniferifuran (reported as amurensin H by some authors), diptoindonesin A (reported as ε-viniferin-C-glucoside by some authors), *trans*-ω-viniferin, *cis*-ω-viniferin, *trans*-δ-viniferin, *cis*-δ-viniferin, a dimethylated derivative of *trans*-ε-viniferin, a dimethylated derivative of *trans*-δ-viniferin, *trans*-scirpusin A, maackin A, a derivative of *trans*-ε-viniferin with γ-lactam ring, a derivative of *trans*-resveratrol with γ-lactam ring, malibatol A, viniferal, vitisinol C, vitisinol E, vitisinol B, viniferether A, and viniferether B (Table 2). It is important to note that there are a number of instances where common names given to particular stilbenoids can lead to confusion. For instance, the name vitisinol E has been given to two different stilbenoid dimers by different authors [8,25,26,27,28,62].

### 5.3. Trimeric Stilbenes

There are eight trimers in *V. vinifera* vegetative organs: ampelopsin B, ampelopsin C, ampelopsin E, *trans*-miyabenol C, *cis*-miyabenol C, davidiol A, α-viniferin, and viniferol D (Table 2) [9,31,56,57,67].

### 5.4. Tetrameric Stilbenes

Among stilbene tetramers, the following 16 compounds are reported in the vegetative organs of grapevine: hopeaphenol, isohopeaphenol, ampelopsin H, vaticanol C-like isomer (or vaticanol C by some authors), vitisin A (r2-viniferin), vitisin B (r-viniferin), vitisifuran A, vitisifuran B, vitisin C, viniferol A, viniferol B, viniferol C, viniferol E, wilsonol C, heyneanol A, and stenophyllol C (reported as napalensinol B by some authors) (Table 2) [7,25,28,29,31,41,67].

### 5.5. Pentameric Stilbenes

Two stilbenes pentamers have been reported in the *Vitis* genus [39]. However, none have been detected in the vegetative organs.

### 5.6. Hexameric Stilbenes

Viniphenol A, a new resveratrol hexamer, is the only hexameric stilbene isolated from *V. vinifera* leaves (Table 2) [26].

## 6. Levels of Phenolic Compounds in the Leaves, Stems, Canes, Woods, and Roots of the Vine Plant

Several phenolic compounds have been quantified in grapevine, although absolute quantification is currently not available for many of them [9,55,59]. Most authors have reported their data on either a fresh or dry weigh basis (Tables S1 and S2). In analyzing the data for this review, mean values were first calculated using fresh and dry weight values separately, and then together. Although the separate analysis proved challenging because of few data points available for many of the compounds, the final ranking of phenolics was not affected by the method of calculation.

On another note, many of the compounds are quantified as equivalents of the most similar chemicals [12,13,51]. Comparison of the calibration curves showed that assays of content determination in vine of stilbenes in which equivalent chemicals are used as standards lead to a severe underestimation of the oligomer concentration. For example, the quantification of ε-viniferin using *trans*-resveratrol as standard underestimated its concentration by a factor > 2 in the study by [24]. Therefore, caution is required when interpreting the data presented in Table 3, Table 4, Table 5, Table 6, Table 7, Table 8, Table 9 and Table 10, taking into consideration the water status of the samples analyzed as reported by the different authors, and the number of studies and data points used in the calculation of mean values (Tables S1 and S2).

### 6.1. Phenolic Compounds in Grapevine Leaves

A total of 132 phenolic compounds have been reported in grapevine leaves (Table 3 and Table 4). 

Eighty-seven phenolic acids and flavonoids, and five coumarins have been identified in the leaves of grapevine, with the highest level recorded for quercetin-3-*O*-glucuronide (10,305.10 mg/kg on average), followed by quercetin-3-*O*-galactoside (7436.94 mg/kg), quercetin-3-*O*-glucoside (7256.42 mg/kg), caftaric acid (4151.97 mg/kg), quercetin-3-*O*-rhamnoside (2708.60 mg/kg), kaempferol-3-*O*-glucoside (1730.09 mg/kg), kaempferol-3-*O*-glucuronide (662.34 mg/kg), coutaric acid (635.81 mg/kg), myricetin-3-*O*-glucoside (291.23 mg/kg), quercetin-3-*O*-rutinoside (257.51 mg/kg), and kaempferol-3-*O*-rutinoside (200.34 mg/kg) (Table 3). Among compounds with levels between 10 and 100 mg/kg are five favan-3-ols (gallocatechin gallate, 49.40 mg/kg; gallocatechin, 40.20 mg/kg; epigallocatechin, 23.77 mg/kg; epigallocatechin gallate, 10.69 mg/kg; and catechin; 10.62 mg/kg), and two phenolic acids (fertaric acid, 85.48 mg/kg; and *p*-hydroxybenzoic acid, 83.40 mg/kg) (Table 3). These levels in general agree with reports that grapevine leaves are rich sources of flavonols. In their studies, [2,20,22,79,84,85] found that the predominant phenolics in the leaves are quercetin-3-*O*-glucuronide, quercetin-3-*O*-glucoside, caftaric acid, and kaempferol-3-*O*-glucoside; total amounts of quercetin derivatives were significantly higher than total amounts of kaempferol derivatives in the studies [2,79,85].

The 40 stilbenes reported in the leaves of *V. vinifera* are eminently less abundant than the phenolic acids and flavonoids. The most predominant stilbenes in the leaves with levels superior to 50 mg/kg are *trans*-resveratrol (137.88 mg/kg), vaticanol C-like isomer (96.85 mg/kg), *cis*-piceid (78.38 mg/kg), *trans*-piceatannol (78.04 mg/kg), ampelopsin H (76.08 mg/kg), α-viniferin (71.61 mg/kg), and *cis*-miyabenol C (50.37 mg/kg) (Table 4). All these compounds are often undetected in healthy leaves. For example, in two grapevine varieties grown in Serbia, the total stilbene content was 45% higher in infected than in healthy leaf extracts [73]. Interestingly, some leaf samples have been found to contain *cis*-resveratrol-3-*O*-glucoside instead of the expected *trans*-resveratrol as their predominant stilbene [59,60,84].

### 6.2. Phenolic Compounds in Grapevine Stems

Literature data have revealed the presence of 88 phenolic compounds (of which 47 were stilbenes) in the stems of grapevine. Although the most abundant compound is the flavonol quercetin-3-*O*-galactoside (17,403.61 mg/kg), flavan-3-ols constitute the majority of compounds in the stems and in the order catechin (14,900.45 mg/kg) > gallic acid (10,307.36 mg/kg) > epicatechin (9251.64 mg/kg) > procyanidin B1 (9216.18 mg/kg) > procyanidin T2 (9100.99 mg/kg) > procyanidin B3 (8724.23 mg/kg) > epicatechin gallate (6362.96 mg/kg) > procyanidin C1 (5007.76 mg/kg) > procyanidin B4 (2243.10 mg/kg) > procyanidin dimer gallate (2234.08 mg/kg) > procyanidin B2 (2056.93 mg/kg) > procyanidin A1 (1254.38 mg/kg) (Table 5). The high level of flavan-3-ols in the stems agrees with several reports [33,34,37,54,69]. Among other compounds with average values superior to 900 mg/kg are the phenolic acid caftaric acid (3373.18 mg/kg) and the flavonols quercetin (4266.04 mg/kg), quercetin-3-*O*-glucoside (1785.38 mg/kg), quercetin-3-*O*-rhamnoside (1570.10 mg/kg), kaempferol (950.35 mg/kg), and quercetin-3-*O*-glucuronide (908.82 mg/kg), which are also abundant compounds in the leaves. In the stems, two anthocyanins are reported with average values superior to 500 mg/kg, namely, malvidin-3-*O*-rutinoside (539.89 mg/kg) and malvidin-3-*O*-glucoside (513.13 mg/kg) (Table 5). In the stems from seven cultivars grown in northern Portugal, caftaric acid, quercetin-3-*O*-glucuronide, malvidin derivatives, and epicatechin were the main metabolites, representing from 54% to 75% of the total phenolic content [33].

Only a few of the 47 stilbenic compounds identified in the stems of grapevine have been quantified [58]: *trans*-resveratrol (506.41 mg/kg), (+)-*trans*-ε-viniferin (433.49 mg/kg), ampelopsin D (65.00 mg/kg), vitisin B (33.95 mg/kg), *trans*-piceid (14.52 mg/kg), *trans*-isorhapontigenin (9.90 mg/kg), *trans*-piceatannol (7.42 mg/kg), and *trans*-δ-viniferin (4.86 mg/kg) (Table 6).

### 6.3. Phenolic Compounds in Grapevine Canes

Compared to leaves and stems, fewer studies have been conducted on the phenolic composition of grapevine canes, which explains the report of only 49 phenolic compounds for the organ. These data indicate that canes have substantial quantities of valuable health-promoting stilbenes [7,12,40,46,51,52,58]. Compounds usually present in the cane extracts Table 7) are *trans*-resveratrol (2797.17 mg/kg), (+)-*trans*-ε-viniferin (2449.25 mg/kg), isohopeaphenol (851.43 mg/kg), vitisin B (668.08 mg/kg), *trans*-piceatannol (583.88 mg/kg), *trans*-ω-viniferin (556.41 mg/kg), and hopeaphenol (511.39 mg/kg); the remaining compounds are with levels below 500 mg/kg.

Other compounds identified in the canes with significant amounts are catechin (1747.01 mg/kg), sinapic acid (1154.81 mg/kg), procyanidin B1 (511.12 mg/kg), epicatechin (269.40 mg/kg), ferulic acid (165.63 mg/kg), gallic acid (165.06 mg/kg), prodelphinidin A-type (160.17 mg/kg), and protocatechuic acid (103.31 mg/kg) (Table 8). Ferulic acid, for example, has been reported as the major compound in the shoots of various grapevine cultivars [47,83].

### 6.4. Phenolic Compounds in Grapevine Woods

No report was found related to the identification of phenolic acids and flavonoids in the woods (trunk and cordons) of the vine plant. All the 23 stilbenes identified in the woods of various cultivars have been quantified, as shown in Table 9, and with generally high levels ranging from 13.28 mg/kg for *trans*-astringin to 8263.87 mg/kg for (+)-*trans*-ε-viniferin [12,13]. The most abundant compounds are (+)-*trans*-ε-viniferin (8263.87 mg/kg), (+)-*cis*-ε-viniferin (3851.97 mg/kg), isohopeaphenol (2429.69 mg/kg), α-viniferin (2366.03 mg/kg), and *trans*-resveratrol (2195.12 mg/kg). Eight compounds are with levels between 1000 and 2000 mg/kg, seven with levels between 100 and 1000 mg/kg, and three with levels between 10 and 50 mg/kg.

### 6.5. Phenolic Compounds in Grapevine Roots

As with the woods, there are no reports on the phenolic acid and flavonoid profiles of grapevine roots. The 24 stilbenes in the roots [12,30,41,42,62] can be ranked, on the basis of abundance, in the following order: (+)-*trans*-ε-viniferin (6475.95 mg/kg) > vitisin B (6420.48 mg/kg) > hopeaphenol (1814.90 mg/kg) > ampelopsin A (1096.92 mg/kg) > vitisin A (1090.12 mg/kg) > isohopeaphenol (529.54 mg/kg) > *trans*-resveratrol (503.25 mg/kg) (Table 10).

## 7. Distribution of Phenolic Compounds in the Vegetative Organs of Grapevine

**Figure 1:**
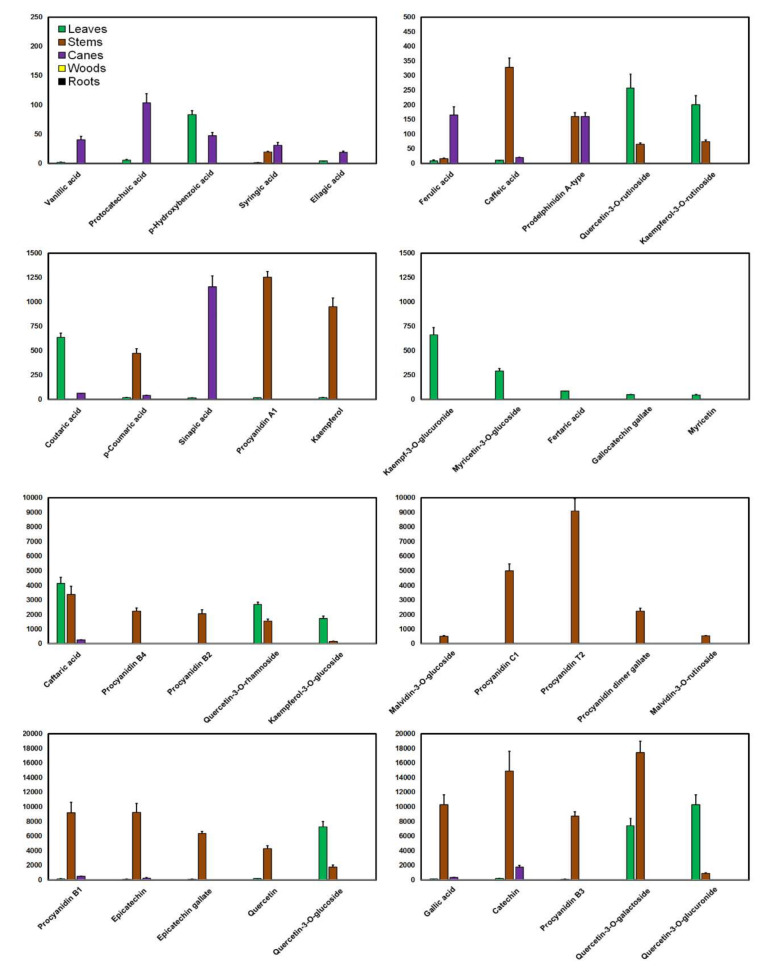
Distribution of the major phenolic acids and flavonoids in the vegetative organs of the vine plant: mean values (*y*-axis; mg/kg) + standard deviations as error bars divided by 10 for better visualization.

**Figure 2:**
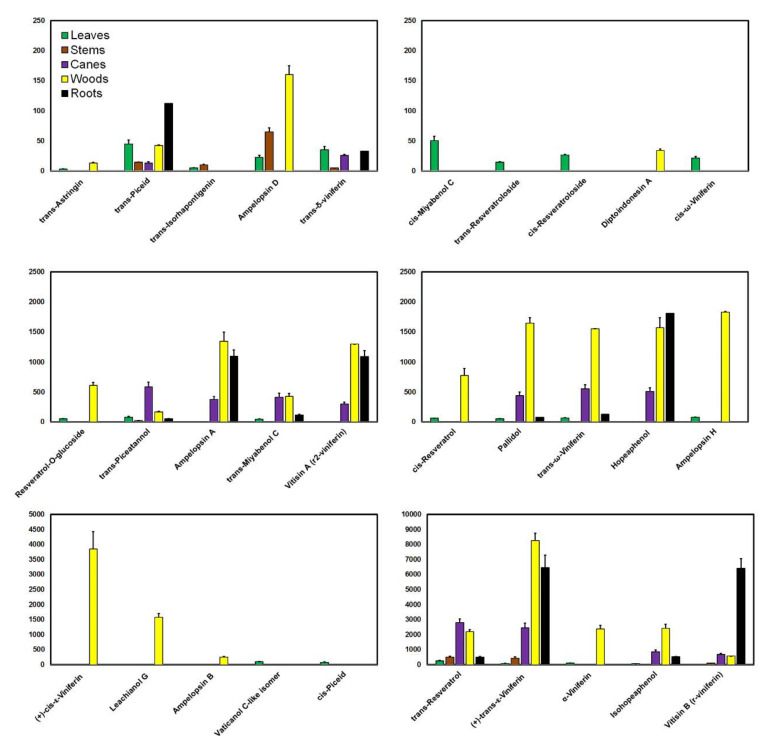
Distribution of the major stilbenic compounds in the vegetative organs of the vine plant: mean values (*y*-axis; mg/kg) + standard deviations as error bars divided by 10 for better visualization.

A mapping of the distribution of phenolic compounds in grapevine shows that composition and levels greatly vary according to the organ investigated (Figure 1 and Figure 2). In general, flavonoids constitute the largest group of phenolics. From an anatomical point of view, these compounds localize specifically in the stems, with lower amounts in the leaves and the canes (Figure 1).

The overall data obtained through adding together the available individual compound means (not shown) indicate that the total amount of phenolics in the stems is on average 114,415.68 mg/kg. Flavan-3-ols constitute the most abundant group of compounds in the stems (61.63%; catechin, epicatechin, and procyanidin B1 with the highest levels), followed by flavonols (23.75%; quercetin-3-*O*-galactoside with the highest level), hydroxybenzoic acids (9.03% with a high abundance of gallic acid), hydroxycinnamic acids (3.66%), anthocyanins (0.99%), and stilbenes (0.94%). From an industrial point of view, stems seem to be an important source of proanthocyanidins for potential use as nutraceutical, enological products, chemical standards, or even in winemaking to regulate the composition of flavonoids in wine [69].

Flavonols are quantitatively the most abundant phenolic class in the leaves (83.43% of the total amount of phenolics, i.e., 37,052.70 mg/kg) with a high abundance of quercetin-3-*O*-glucuronide, quercetin-3-*O*-galactoside, and quercetin-3-*O*-glucoside; flavonols are followed by hydroxycinnamic acids (13.19%, of which caftaric acid and coutaric acid have the highest levels), stilbenes (2.63%), flavan-3-ols (0.41%), and hydroxybenzoic acids (0.26%). Coumarins, flavones, anthocyanins, and flavanones are found in minor amounts. The spatial distribution of phenolic compounds in grapevine leaves evaluated by matrix-assisted laser desorption/ionization (MALDI) showed the specific colocation of *trans*-resveratrol, pterostilbene, and viniferins around the veins in healthy leaves [68]. It is reported that the leaf blade is more abundant in phenolic compounds than the petiole, and much less than the pedicel [75,84].

There are very few reports on flavonols, flavones, flavanones, anthocyanins, and coumarins in the canes. Phenolic groups identified (on average 14,477.42 mg/kg) can be classified in terms of abundance as stilbenes (69.00% of the total) > flavan-3-ols (18.56%; catechin, procyanidin B1 and epicatechin with the highest levels as with the stems) > hydroxycinnamic acids (9.63% of which sinapic acid and ferulic acid) > hydroxybenzoic acids (2.80% of which gallic acid). Indeed, in the comparison of phenolics in the skins, pulps, seeds, canes, and leaves of six cultivars grown in Iran, it was found that the canes usually contained the highest amounts of flavonoids and stilbenes [87].

The data also show that stilbenes accumulate primarily in the woods (34,390.90 mg/kg; ε-viniferin, isohopeaphenol, *trans*-resveratrol, α-viniferin, and ampelopsin H with the highest levels), followed by the roots (18436.44 mg/kg; ε-viniferin, hopeaphenol, vitisin B, and ampelopsin A with the highest levels), the canes (9989.50 mg/kg; ε-viniferin, *trans*-resveratrol, isohopeaphenol, vitisin B, and *trans*-piceatannol with the highest levels), and the stems (1075.55 mg/kg; ε-viniferin, *trans*-resveratrol, vitisin B, and ampelopsin D with the highest levels), whereas leaves, which are more exposed to environmental stresses [23,40], have a low concentration of these compounds (972.97 mg/kg; *trans*-resveratrol, vaticanol C-like isomer, piceid, *trans*-piceatannol, and ampelopsin H with the highest levels) (Figure 2).

Stilbenes are more constitutively expressed in the woods, roots, canes, and stems, where they are believed to help in the prevention of wood rot [12,39,62]. This constitutive expression might explain the more consistent and greater levels of compounds in these plant parts, in contrast to the leaves in which stilbene production is mostly induced to provide enhanced protection against pests and diseases [12,23,65,85]. Levels of stilbenes were compared in the wood, roots, and canes by [12]; grapevine canes usually had ε-viniferin and *trans*-resveratrol as their main compounds, and woods had more ε-viniferin and isohopeaphenol, whereas roots were generally rich in vitisin B, ampelopsin A, and vitisin A [12]. The authors concluded that the degree of oligomerization of stilbenes increases from the aerial organs to the root system. After manual dissection of the cortex, pith, and conducting tissues of grape canes, the evaluation of the spatial distribution of stilbenes suggested a predominance of monomers in conducting tissues and oligomers in cortex and pith [52].

## 8. Factors Affecting the Biosynthesis and Levels of Phenolic Compounds in the Vegetative Organs of Grapevine

Grapevine phenolics occur in large concentration ranges, as attested by standard deviation values presented in Table 3, Table 4, Table 5, Table 6, Table 7, Table 8, Table 9 and Table 10. There are a number of factors that interact together, so as to result in such wide range of phenolic variations. Some of these factors are well documented in the literature and include cultivars, climate, cultural practices, and biotic and abiotic stresses.

### 8.1. Grapevine Cultivars and Rootstocks

Grapevine cultivars are not genetically homogeneous, and most of them are multiplied by vegetative propagation. A collection of vines propagated from the same mother vine make up a clone; clonal selection is routinely carried out in viticulture with the purpose of creating disease-free or high-yielding populations. Progressively, criteria such as the levels of grape sugar and skin phenolic compounds have been integrated in clonal selection programs. Moreover, these clones are often grafted on different rootstocks [2,9,90]. These factors could explain why the contents of polyphenols are subject to such severe variations [6,32,58,59,89]. For instance, plants of Pinot blanc grafted on three different rootstocks—Kober 5B, S04, and 1103P—accumulated resveratrol differentially in the leaves [14]. According to some authors, the best resveratrol-producing cultivars are Pinot noir and Cabernet Sauvignon, depending on the clones investigated [5,6,51]. A comparison of the phenolic profile of canes of the cultivars Chardonnay, Cabernet Sauvignon, Shiraz (Syrah), Merlot, Sauvignon blanc, and Pinot noir showed that Pinot noir had very high levels of *trans*-resveratrol and *trans*-ε-viniferin [51]. In several studies, quantitative analyses showed that the stems and leaves of red cultivars are richer in proanthocyanidins, flavonols, hydroxycinnamic acids, anthocyanins, and stilbenes than those of white cultivars [33,53,54,91]. The biosynthesis of phenolic compounds is also closely dependent on plant developmental stages. It has indeed been reported that very young and very old leaves do not synthesize high stilbene and anthocyanin levels, probably because of the incomplete development of the stomata [9,69,77,79,84]. In the study by [89], however, the intensity of stilbene induction did not show a clear and homogeneous correlation with the position of leaves along the shoot. The total viniferin content was generally highest in the second, third, and fourth leaves for the 21/103 genotype. Moreover, [25] did not find a homogeneous trend of change in stilbenoid levels in the stems during the growth cycle.

### 8.2. Geographical Location and Climate (Shading, Temperature, Irrigation)

The geographical location of the vineyard (especially latitude and elevation) and the seasonal meteorological variability in the area are known to influence the phenolic composition of grapevine organs. Meteorological variability, including light, temperature, and water, represents one of the main environmental factors responsible for phenolic biosynthesis. Clear separation was demonstrated between the phenolic profile of leaves [32,79,86] and stems [40] of grapevine cultivars of different geographical origins. A higher variation of stilbene levels between years as compared to variation between plants of the same year have also been reported [89]. Different light exposures of the vine demonstrated that shading decreases the flavonoid content of the leaves, a result that is consistent with the role these molecules play in protecting tissues from UV light [85,91]. In the study by [59], accumulation of quercetin-3-*O*-glucoside, kaempferol-3-*O*-glucoside, and quercetin-3-*O*-galactoside was the most prominent in full sunlight-exposed leaves compared with half-shaded leaves. Furthermore, the biosynthesis of phenolics is sensitive to diurnal differences in temperature, although with different temporal patterns. Indeed, a decrease of flavonoid biosynthesis has been observed when the temperature is limiting or excessive [92]. Extreme weather conditions with prolonged dry periods as well as heavy rain events can severely influence grapevine physiology [2,86]. Water deficit has been reported to upregulate the expression of genes of the anthocyanin pathway [77] and to increase the levels of most polyphenols in the leaves, in particular *cis*-resveratrol-3-*O*-glucoside, kaempferol-3-*O*-glucoside, and quercetin-3-*O*-glucoside [60]. Recent results show that, during water stress, the synthesis of anthocyanins is paralleled by an increase of the expression of flavonoid transporters [65]. On the other hand, it is reported that excessive water application could induce a decrease in proanthocyanidin levels [92].

### 8.3. Vinicultural Practices

There are many cultural practices that affect the production and accumulation of phenolic compounds in grapevine organs. However, many of these factors seem to act in a typical bell-shaped manner, where they could improve the final levels of compounds only when present at optimal levels. For instance, pruning greatly influences the levels of stilbenoids in the canes, leaves, and stems of grapevine, but the effect depends on the number of branches removed and the duration of the treatment [40,50]. In canes remaining on the plant 30 days after pruning, only a minor increase of total stilbenoid levels was observed, whereas in canes stored at room temperature after pruning, a twofold increase occurred [40]. In general, practices that increase plant vigor, such as fertilizer application, are reported to negatively influence the biosynthesis of phenolics in grapevine. Less *trans*-resveratrol was accumulated in the leaves when increasing nitrogen doses were applied to one-year-old potted vines, whereas the opposite was observed with increased potassium doses; when nitrogen and potassium were supplied together, potassium did not balance the negative effect of nitrogen [14]. Iron deficiency stimulated anthocyanin accumulation in grapevine apical leaves [78]. The application of other agrochemicals (e.g., plant hormones and chitosan) with the aim of enhancing vegetative growth and grape quality also affects phenolic biosynthesis in a complex way [18]. Typically, it has been reported that abscisic, auxin, and ethylene application lead to an increase in the levels of flavonoids, whereas the opposite has been observed with the application of gibberellic acid and inhibitors of the ethylene receptor [65]. Indirectly, fertilizer and hormone application could also lead to low levels of polyphenols because they induce the production of especially dense foliage that limits the exposure of some organs to sunlight. Grapevine is susceptible to various pests and diseases usually controlled by chemical and biological treatments that can introduce additional variability in the data. High amounts of stilbenoids were produced in grapevine plants that were mycorrhized with *Rhizophagus irregularis,* as well as an up-regulation in the leaves of genes involved in the stilbene biosynthesis pathway [70].

### 8.4. Outside and Biotic Stimuli

High variability in phenolic levels in grapevine is best explained by biotic stresses and mechanical injuries. In most studies, it was found that the infection status of the plant influences phenolic profiles much more than other factors [9]. Indeed, following pathogen attacks and insect bites, all the vegetative organs of grapevine undergo modifications in terms of their polyphenol composition and contents. For phenolic acids, flavonoids, and coumarins, the literature is contradictory regarding the relationship between level and disease susceptibility [15]. This might be due to the fact that these compounds are part of the constitutive metabolome in lignified tissues. In the study by [77], the expression of flavonoid pathway genes was detected in both healthy and diseased leaves, confirming that the pathway is active in control conditions [77]. These polyphenols that are present prior to an attempted infection of the plant are known as preformed, and are part of a passive resistance mechanism [14,36]. A mechanism of active resistance is the synthesis, degradation, or metabolism to a different compound in response to attacks by pathogens; de novo synthesized compounds are called phytoalexins [71]. An induction in the synthesis of stilbenic compounds in photosynthetic tissues has been considerately reported in response to the main grapevine pathogens, namely, *Botrytis cinerea* of grey mould [70], *Plasmopara viticola* of downy mildew [6,10,12,52,61,62,64,68,70,73,82,89], *Erysiphe necator* of powdery mildew [11,88], fungi associated with grapevine trunk diseases [3,13,15,43,44,45,86], *Rhizopus stolonifera* of berry rot [14], *Grapevine leafroll-associated virus 3* of Grapevine leafroll disease [77], *Xylella fastidiosa* of Pierce’s disease [9,55], and *Aspergillus carbonarius* of sour rot [71]. The increment can be as high as 100-fold, and has a biosynthetic origin attributable to stilbene synthase induction. Interestingly, it was found that downy mildew affects the spatial repartition of stilbenoids in the cane, with an increase in the cortex (a tissue notably involved in protection against mechanical damage and microbial attack) and conducting tissues, and a decrease in the pith [52]. Mechanical stress on freshly pruned canes and leaves have also been reported to overinduce the biosynthesis of *trans*-resveratrol and *trans*-piceatannol within a short period after pruning [23,40].

## 9. Concluding Remarks

Several compounds with phenolic characteristic have been detected in the leaves, stems, canes, woods, and roots of the grapevine plant. An effort is still needed to identify and quantify several of these compounds. It is clear that several factors affect the biosynthetic pathways, leading to the accumulation of phenolic compounds in grapevine. The patterns of gene expression show significant differences between organs and cultivars, especially for genes involved in stilbene synthesis. In the leaves in particular, random inductions in the synthesis of these compounds have been observed, which is understandable given their higher exposure to the environment and resulting susceptibility to attack by pests and diseases. An understanding of the different roles of these factors is crucial because only with this information will it be possible to develop cultural practices aimed at improving phenolic levels in the plants and in the derived products. Moreover, unexplored areas of research related to this topic will most certainly constitute a basis for future improvement of grapevine disease tolerance.

## Figures and Tables

**Table 1:** Polyphenols (excluding stilbenes) in the vegetative organs of the grapevine plant.

ID ^1^	Compound Group	Compound Name ^1^	Chemical Formula ^2^	MW (g/mol)	[M–H]–	Main MS/MS Fragments (m/z) ^3,4^	λmax (CH_3_OH) (nm)	Detection Mode ^5^	Tissue Distribution ^6^				
									LEAVES	STEMS	CANES	WOODS	ROOTS
1	Hydroxybenzoic acid	Quinic acid	C7H12O6	192.167	191	**111**, **173**, 85, 127	308, 280	MS	✓				
2	Hydroxybenzoic acid	Gallic acid	C7H6O5	170.120	169	**125**, 124, 79, 51	278, 214	MS, NMR, DAD	✓	✓	✓		
4	Hydroxybenzoic acid	Protocatechuic acid	C7H6O4	154.121	153	**109**, 108	297, 258	MS	✓		✓		
7	Hydroxybenzoic acid	*p*-Hydroxybenzoic acid	C7H6O3	138.122	137	**93**, 60, 79, 108, 118, 137	272, 310sh	MS	✓		✓		
8	Hydroxybenzoic acid	Gentisic acid	C7H6O4	154.121	153	**109**, 81, 42, 108	281, 228, 330sh	MS	✓				
9	Hydroxybenzoic acid	γ-Resorcylic acid	C7H6O4	154.121	153	**109**, **136**, **154**, 110, 52, 80, 137, 39, 155	313, 245	MS	✓				
30	Hydroxybenzoic acid	Vanillic acid	C8H8O4	168.149	167	**123**, **152**, **108**, 91	292, 260	MS, NMR, DAD	✓		✓		
36	Hydroxybenzoic acid	Syringic acid	C9H10O5	198.174	197	**182**, **153**, **167**, 138	276	MS, NMR, DAD	✓	✓	✓		
50	Hydroxybenzoic acid	Ellagic acid	C14H6O8	302.194	301	**284**, 300, 257, 229, 184	367, 256, 301sh	MS	✓		✓		
14	Hydroxycinnamic acid	1-*O*-Sinapoyl-*β*-D-glucose	C17H22O10	386.353	385	**223**, 205, 341, 265, 190, 179, 119, 247	282	MS	✓				
16	Hydroxycinnamic acid	1-*O*-(4-Coumaroyl)-glucose	C15H18O8	326.301	325	**163**, **145**, **119**, 187, 265, 205	322	MS	✓	✓			
20	Hydroxycinnamic acid	1-Caffeoyl-*β*-D-glucose	C15H18O9	342.300	341	**179**, **161**, 143, 149, 131, 135	290, 304sh, 328	MS	✓	✓			
21	Hydroxycinnamic acid	Ferulic acid pentose	NA	NA	325	**149**, 178, 193	326, 275	MS	✓	✓			
22	Hydroxycinnamic acid	Caftaric acid isomer	C13H11O9	311.224	311	**179**, 135, 149	325, 286	MS	✓				
24	Hydroxycinnamic acid	Caftaric acid	C13H12O9	312.230	311	**179**, **135**, **149**, 267, 161, 237	326, 298sh, 243	MS, NMR, DAD	✓	✓	✓		
27	Hydroxycinnamic acid	Coutaric acid	C13H12O8	296.231	295	**163**, **149**, **119**	316, 234, 300sh	MS, DAD	✓		✓		
28	Hydroxycinnamic acid	Chlorogenic acid	C16H18O9	354.311	353	**191**, **179**, 135, 161, 335, 172, 284	328, 244, 303sh	MS, DAD	✓				
31	Hydroxycinnamic acid	Chicoric acid	C22H18O12	474.374	473	**311**, **293**, **179**, 149, 135, 219	328, 305sh, 279	MS		✓			
32	Hydroxycinnamic acid	Fertaric acid	C14H14O9	326.259	325	**193**, **175**, **149**, 281, 134	314, 279	MS	✓				
35	Hydroxycinnamic acid	Caffeic acid	C9H8O4	180.159	179	**135**, 134, 96	324, 299sh, 240	MS, NMR, DAD	✓	✓	✓		
43	Hydroxycinnamic acid	*p*-Coumaric acid	C9H8O3	164.160	163	**119**, **104**, 93	310, 225, 211, 310sh	MS, NMR, DAD	✓	✓	✓		
46	Hydroxycinnamic acid	Ferulic acid	C10H10O4	194.186	193	**134**, **149**, 178, 116	323, 289, 238sh	MS, NMR, DAD	✓	✓	✓		
48	Hydroxycinnamic acid	Sinapic acid	C11H12O5	224.212	223	**164**, 149, 208, 164, 193, 179	318, 238	MS	✓		✓		
83	Hydroxycinnamic acid	Cinnamic acid	C9H8O2	148.161	147	103, 77, 87, 129	276, 215, 203	DAD	✓				
3	Flavan-3-ol	Gallocatechin	C15H14O7	306.270	305	**179**, **221**, **219**, 165, 261, 125, 137	274, 370	MS	✓				
6	Flavan-3-ol	Procyanidin B1	C30H26O12	578.526	577	**425**, **407**, 289, 451, 287, 245, 451	275, 322	MS, NMR, DAD	✓	✓	✓		
10	Flavan-3-ol	Procyanidin A1	C30H24O12	576.501	575	**449**, **289**, 423, 539, 477, 407, 441	280	MS	✓	✓	✓		
15	Flavan-3-ol	Epigallocatechin	C15H14O7	306.270	305	**179**, **165**, **219**, **221**, 261, 125, 261, 125	274, 212, 235	MS	✓				
18	Flavan-3-ol	Procyanidin C1	C45H38O18	866.778	865	**695**, **407**, 577, 287, 713, 739, 575, 425, 289	279	MS, DAD		✓	✓		
19	Flavan-3-ol	Procyanidin T2	C45H38O18	866.778	865	**577**, **713**, 289, 287, 425, 575, 695, 407, 739	280	MS, DAD		✓	✓		
23	Flavan-3-ol	Catechin	C15H14O6	290.271	289	**245**, **203**, **179**, 205, 227, **109**, **123**, 165, 125, 151	275, 222	MS, NMR, DAD	✓	✓	✓		
25	Flavan-3-ol	Procyanidin B3	C30H26O12	578.526	577	**425**, **407**, 289, 151, 559, 445	270, 330	MS, NMR, DAD	✓	✓			
26	Flavan-3-ol	Procyanidin B4	C30H26O12	578.526	577	**425**, **407**, 289	280, 240	MS, DAD	✓	✓			
29	Flavan-3-ol	Procyanidin B2	C30H26O12	578.526	577	**425**, **407**, 289, 451, 287, 245, 125	280, 240, 370	MS, NMR, DAD	✓	✓	✓		
37	Flavan-3-ol	Epigallocatechin gallate	C22H18O11	458.375	457	**305**, **219**, 261, 221, 359, 169, 305, 289, 271, 125, 331	274, 238	MS, DAD	✓				
38	Flavan-3-ol	Prodelphinidin A-type	C30H26O13	594.527	593	**425**, **441**, **573**, 423, 407, 289, 531, 273, 339, 245, 177, 161	276, 228, 320	MS		✓	✓		
39	Flavan-3-ol	Procyanidin dimer gallate	NA	NA	729	**577**, **407**, **559**, 451, 711, 289, 593, 437, 425	280	MS, DAD		✓	✓		
40	Flavan-3-ol	Epicatechin	C15H14O6	290.271	289	**245**, **203**, **109**, **179**, 205, 123, 125, 151	277, 226	MS, NMR, DAD	✓	✓	✓		
42	Flavan-3-ol	Gallocatechin gallate	C22H18O11	458.375	457	**169**, **161**, **359**, 331, 169, 305, 193	276, 240	MS	✓				
47	Flavan-3-ol	Epicatechin gallate	C22H18O10	442.376	441	**289**, **245**, 205, **169**, **125**, 331, 271, 179	278, 240	MS, NMR, DAD	✓	✓			
61	Flavan-3-ol	Catechin gallate	C22H18O10	442.376	441	**289**, **245**, **205**, 331, 169, 125, 425, 271, 395, 169, 193, 405	278	MS	✓	✓			
33	Anthocyanin	Delphinidin-3-*O*-glucoside	C21H21O12+	465.387	463	**301**, 300, 271, 125	526, 361, 277, 402sh	DAD	✓				
34	Anthocyanin	Cyanidin-3-*O*-glucoside	C21H21O11+	449.388	447	**284**, **211**, 285, 255, 147, 227	516, 262, 301sh	MS, DAD	✓				
44	Anthocyanin	Petunidin-3-*O*-glucoside	C22H23O12+	479.414	477	314, 315, 299, 300	526, 344sh, 277	DAD	✓				
45	Anthocyanin	Peonidin-3-*O*-glucoside	C22H23O11+	463.415	461	**299**, 298, 284, 255, 227, 211	517, 280, 330sh, 421sh	MS, DAD	✓				
52	Anthocyanin	Malvidin-3-*O*-glucoside	C23H25O12+	493.441	491	**329**, 314, 299	528, 348sh, 288	MS, DAD	✓	✓			
59	Anthocyanin	Petunidin-3-(6-*O*-acetyl)glucoside	C24H25O13+	521.451	519	**315**, 302, 274, 149	528, 270, 350sh	DAD	✓				
62	Anthocyanin	Peonidin-3-(6-*O*-acetyl)glucoside	C24H25O12+	505.452	504	***301*** *, 286, 230, 258, 268*	522, 280	DAD	✓				
67	Anthocyanin	Malvidin-3-(6-*O*-acetyl)glucoside	C25H27O13+	535.478	533	**329**, 315	522, 344, 278	DAD	✓				
72	Anthocyanin	Cyanidin-3-(6-*O*-coumaroyl)glucoside	C30H27O13+	595.533	593	***287*** *, 259, 231, 213, 259*	524, 314, 284, 449sh	DAD	✓				
80	Anthocyanin	Petunidin-3-(6-*O*-coumaroyl)glucoside	C31H29O14+	625.553	624	***317*** *, 302, 274, 218, 228, 246*	534, 282, 313	DAD	✓				
81	Anthocyanin	Peonidin-3-(6-*O*-coumaroyl)glucoside	C31H29O13+	609.554	608	***301*** *, 286, 230, 258, 268*	522, 312	MS, DAD	✓				
85	Anthocyanin	Malvidin-3-(6-*O*-coumaroyl)glucoside	C32H31O14+	639.586	637	**329**, 299, 281	534, 318	DAD	✓				
86	Anthocyanin	Malvidin-3-(6-*O*-caffeoyl)glucoside	C32H31O15+	655.581	655	**331**, 299, 637, 315, 475	532, 324, 284	MS		✓			
87	Anthocyanin	Malvidin-3-*O*-rutinoside	C29H35O16+	639.583	637	**331**	526, 288	MS		✓			
41	Flavanone	Taxifolin	C15H12O7	304.254	303	**285**, **125**, **177**, 275, 151, 259, 217	290, 326sh	MS	✓				
55	Flavanone	Taxifolin-*O*-pentoside	C20H20O11	436.371	435	**303**, **285**, **399**, 151, 241, 217, 175	274, 317	MS		✓			
56	Flavanone	Taxifolin-3-*O*-glucoside	C21H22O12	466.395	465	**285**, 303, 151, 339, 177, 259, 447	290	MS		✓			
57	Flavanone	Taxifolin-3-*O*-rhamnoside	C21H22O11	450.396	449	**303**, **285**, 151, 323, 431	292, 235	MS		✓			
88	Flavanone	Hesperetin	C16H14O6	302.282	301	**258**, **143**, **157**, 137, 286	284, 324sh, 221	MS	✓				
95	Flavanone	Eriodictyol-7-*O*-glucoside	C21H22O11	450.396	449	**287**, **269**, **151**, 135, 259, 225, 209, 431	281, 327	MS	✓				
103	Flavanone	Naringenin	C15H12O5	272.256	271	**151**, **177**, **119**, 165, 125, 107, 227, 191	289, 228, 336sh	MS	✓				
104	Flavanone	Naringenin-7-*O*-glucoside	C21H22O10	434.397	433	**271**, 269, 313, 177, 151, 119, 107	282, 222	MS	✓				
49	Flavonol	Myricetin-3-*O*-galactoside	C21H20O13	480.378	479	**317**, **316**, 178, 271	360, 265	MS, DAD	✓				
51	Flavonol	Myricetin-3-*O*-glucuronide	C21H18O14	494.361	493	**317**	353, 300sh, 261	MS, DAD	✓				
53	Flavonol	Myricetin-3-*O*-glucoside	C21H20O13	480.378	479	**317**, 316, 169, 271, 303, 227, 179, 151	362, 298sh, 260	MS, DAD	✓				
54	Flavonol	Quercetin-3-*O*-rutinoside	C27H30O16	610.521	609	**301**, **300**, **271**, **255**, 179, 343, 151	353, 256, 294sh	MS, NMR, DAD	✓	✓			
58	Flavonol	Quercetin-3-*O*-galactoside	C21H20O12	464.379	463	**301**, **300**, 179, 273, 257, 151	362, 256, 301sh	MS, NMR, DAD	✓	✓	✓		
60	Flavonol	Quercetin-3-*O*-glucoside	C21H20O12	464.379	463	**301**, **300**, **271**, 161, 179, 255, 151	358, 256, 300sh	MS, NMR, DAD	✓	✓			
63	Flavonol	Quercetin-3-*O*-glucuronide	C21H18O13	478.362	477	**301**, **179**, 151, 283, 459, 431, 501	356, 254, 300sh	MS, NMR, DAD	✓	✓			
65	Flavonol	Myricetin-3-*O*-rhamnoside	C21H20O12	464.379	463	**317**, **316**, **271**, 300, 179, 287, 151	372, 302sh, 248	MS	✓				
68	Flavonol	Myricetin	C15H10O8	318.237	317	**151**, **179**, 137, 287, 271, 109, 192	372, 253, 303sh, 207	MS, NMR, DAD	✓				
69	Flavonol	Quercetin-3-*O*-rhamnoside	C21H20O11	448.380	447	**301**, **300**, **179**, 151, 271, 257	354, 258, 307sh	MS, NMR, DAD	✓	✓			
70	Flavonol	Kaempferol-3-*O*-galactoside	C21H20O11	448.380	447	**285**, 255, 227, 327	361, 260	MS	✓				
71	Flavonol	Kaempferol-3-*O*-rutinoside	C27H30O15	594.522	593	**285**, **257**, 151, 447, 199, 241, 93	354, 274	MS, DAD	✓	✓			
73	Flavonol	Kaempferol-3-*O*-glucuronide	C21H18O12	462.363	461	**285**, 267, 443, 417, 257, 229	348, 265	MS, DAD	✓				
74	Flavonol	Quercetin-3-(6-*O*-acetyl)glucoside	C23H22O13	506.416	505	**301**, 300, 463, 271, 255	354, 256, 267sh, 298sh	MS	✓				
75	Flavonol	Quercetin-3-(3-*O*-arabinosyl)glucoside	C26H28O16	596.493	595	**301**, **517**, 151, 300, 463, 179, 445, 271, 255	354, 260, 231	MS	✓				
76	Flavonol	Quercetin-3-(7-O-glucosyl)glucuronide	C27H28O18	640.503	639	**477**, **301**, 179, 151	361, 300, 268, 256	DAD	✓				
77	Flavonol	Quercetin-3-*O*-arabinose	C20H18O11	434.350	433	**301**, **179**, 151, 300, 283	358, 311	MS		✓	✓		
78	Flavonol		C33H40O21	772.662	771	609, 301	355, 259, 299sh, 204	NMR, DAD	✓				
79	Flavonol	Kaempferol-3-*O*-glucoside	C21H20O11	448.380	447	**285**, **255**, 151, 227, 327, 243	348, 263, 297sh	MS, DAD	✓	✓			
82	Flavonol	Quercetin	C15H10O7	302.239	301	**151**, **179**, 273, 193, 257, 229	372, 255, 202sh, 300sh	MS, NMR, DAD	✓	✓			
84	Flavonol	Kaempferol	C15H10O6	286.239	285	**187**, **117**, **211**, 127, 257, 151, 169, 241	369, 258, 390	MS, NMR, DAD	✓	✓			
89	Flavonol	Kaempferol-3-*O*-xyloside	C20H18O10	418.354	417	**285**, 255, 227	350	MS	✓				
90	Flavonol	Kaempferol-3-*O*-rhamnoside	C21H20O10	432.381	431	**285**	351, 264, 202, 294sh	MS	✓				
91	Flavonol	Dihydrokaempferol-3-*O*-rhamnoside	C21H22O10	434.397	433	**269**, **287**, 259, 180, 151, 368	286, 230	MS		✓			
92	Flavonol	Isorhamnetin-3-*O*-galactoside	C22H22O12	478.406	477	**315**,.314 271, 300, 357	366, 289, 259	MS	✓				
93	Flavonol	Isorhamnetin-3-*O*-glucoside	C22H22O12	478.406	477	**315**, 314, 285, 357, 271	354, 265sh	MS, DAD	✓				
94	Flavonol	Quercetin-3-(6-*O*-rhamnosyl)galactoside	C27H30O16	610.517	609	**301**, **541**, **463**, 300, 271, 255, 179, 447, 151	356, 256, 300	NMR, DAD	✓				
96	Flavonol	Isorhamnetin-3-*O*-arabinose	C21H20O11	448.381	447	**315**, 314, 271, 243	345, 258	MS	✓				
97	Flavonol	Isorhamnetin-3-*O*-glucuronide	C22H20O13	492.389	491	**315**, 255, 151	355, 265sh	MS	✓				
98	Flavonol	Isorhamnetin-3-*O*-rutinoside	C28H32O16	624.548	623	**315**, 300, 468	354, 256	MS	✓				
99	Flavonol	Isorhamnetin-3-(6-*O*-feruloyl)glucoside	C32H30O15	654.577	653	**315**	320, 274	MS		✓			
100	Flavonol	Isorhamnetin-3-(4-*O*-rhamnosyl)rutinoside	C34H42O20	770.685	769	461, 623, 163	354, 256	MS, DAD	✓				
101	Flavonol	Kaempferol-3-(6-*O*-coumaroyl)glucoside	C30H26O13	594.525	593	**285**, 227, 255	317, 265, 356sh, 310sh	MS	✓				
102	Flavonol	Kaempferol-3 (7-O-glucosyl)galactoside	C27H30O16	610.521	609	**447**, 489, 285	343, 300sh, 265	DAD	✓				
105	Flavonol	Diquercetin-3-(3-*O*-glucosyl)glucuronide	C42H36O24	924.722	923	765, 755, 837, 903, 935, 808	374	MS	✓				
64	Flavone	Apigenin-7-*O*-glucoside	C21H20O10	432.381	431	**269**, 283, 311, 413, 231, 225, 201, 197, 149	335, 269, 253	MS, NMR, DAD	✓				
66	Flavone	Luteolin-7-*O*-glucoside	C21H20O11	448.380	447	**285**, 226, 257, 217, 241, 198	349, 254sh, 205	MS, NMR, DAD	✓				
5	Coumarin	Aesculin	C15H16O9	340.282	339	**133**, 177, 150	346, 289	MS	✓				
11	Dihydrochalcone	Phlorizin	C21H24O10	436.413	435	**273**, **167**, **229**, 297	285, 230sh	MS	✓				
12	Coumarin	Fraxin	C16H18O10	370.310	369	**207**, **192**, **354**, 149, 123	332, 308sh	MS	✓				
13	Coumarin	Aesculetin	C9H6O4	178.143	177	**149**, **133**, 105, 91, 115, 89, 65	334, 288sh	MS	✓				
17	Coumarin	Umbelliferone	C9H6O3	162.144	161	**133**, 117, 105, 51, 78	323, 236	MS	✓				

^1^ Other reported names are found in Table S1, where compounds are numbered (ID) according to their elution patterns. ^2^ NA = not available or not applicable, MW = Molecular Weight. ^3^ MS-MS values in italic (compounds 62, 72, 80, 81) are reported in positive mode. ^4^ The most abundant fragments are highlighted in bold. ^5^ MS = mass spectrometry detection, NMR = nuclear magnetic resonance detection, DAD = diode array or ultraviolet detection. ^6^ In blue with √ are detected compounds; in light red are undetected compounds or unavailable information.

**Table 2:** Stilbenic compounds in the vegetative organs of the grapevine plant.

ID ^1^	Compound Group	Compound Name ^1,2^	Chemical Formula ^3^	MW (g/mol)	[M–H]– Precursor Ion	Main MS/MS Fragments (m/z) ^4^	λmax (CH_3_OH) (nm)	Detection Mode ^5^	Tissue Distribution ^6^				
									LEAVES	STEMS	CANES	WOODS	ROOTS
106	Monomers	*trans*-Astringin	C20H22O9	406.383	405	**243**, **225**, **201**, 322, 159, 199, 173	331, 305	MS	✓	✓		✓	
107	Monomers	*trans*-Resveratroloside	C20H22O8	390.388	389	**227**, **185**, **179**, 269, 143, 305, 371, 209	311	MS	✓	✓			
108	Monomers	*trans*-Resveratrol-2-*C*-glucoside	NA	NA	389	269, 241, 299, 175, 163	326	MS, NMR		✓	✓		
109	Monomers	*trans*-Resveratrol-10-*C*-glucoside	NA	NA	435	389, 227	315	MS, NMR		✓			
110	Monomers	*trans*-Resveratrol-*O*-glucoside	C20H22O8	390.388	389	**227**, 305, 175, 185	321	MS, NMR		✓	✓	✓	
183	Monomers	*cis*-Resveratrol-*O*-glucoside	C20H23O8	391.391	389	**227**	319, 306	MS	✓				
113	Monomers	*trans*-Piceid	C20H22O8	390.383	389	**227**, **185**, **251**, 269, 209, 371, 143	318, 306, 229	MS, NMR, DAD	✓	✓	✓	✓	✓
117	Monomers	*cis*-Astringin	C20H22O9	406.383	405	**243**, **225**, **201**, 322, 405, 159, 228, 157	324, 260	MS	✓				
119	Monomers	*trans*-Piceatannol	C14H12O4	244.246	243	**175**, **225**, 149, 215, 201, 159, 181, 132, 199, 143	325, 290, 306	MS, NMR	✓	✓	✓	✓	✓
120	Monomers	*cis*-Resveratroloside	C20H22O8	390.388	389	227, 371, 209	280	MS	✓				
121	Monomers	*cis*-Piceid	C20H22O8	390.383	389	**227**, 269, 241, 185, 209, 371, 143	284, 230	MS, NMR	✓		✓		✓
122	Monomers	*trans*-Isorhapontin	C21H24O9	420.411	419	**257**, **241**, 225, 175, 242, 201, 159, 281, 132	326, 303, 290	MS	✓				
123	Monomers	*trans*-Resveratrol	C14H12O3	228.247	227	**185**, **143**, 183, 159, 157, 212, 205	306, 319, 228	MS, NMR, DAD	✓	✓	✓	✓	✓
124	Monomers	2,4,6-Trihydroxyphenanthrene-2-*O*-glucoside	C20H20O8	388.372	389	371, 353, 335, 227, 209, 199	261, 222	MS	✓				
126	Monomers	*trans*-Isorhapontigenin	C15H14O4	258.270	257	**242**, **241**, 224, 172, 213, 185	325, 303, 290	MS	✓	✓	✓		
127	Monomers	*trans*-Pinostilbene-4′-*O*-glucoside	C21H24O8	404.410	403	**241**, 226, 225	NA	MS	✓				
128	Monomers	*cis*-Resveratrol	C14H12O3	228.247	227	**185**, 159, 143, 157, 212, 143	285, 232	MS, NMR	✓			✓	
145	Monomers	*trans*-Pterostilbene	C16H16O3	256.296	255	**239**, 197, 209, 226, 165	298, 305, 275	MS, NMR, DAD	✓	✓			
146	Monomers	*cis*-Pterostilbene	C16H16O3	256.296	255	197, 239, 209, 226, 165	279	MS	✓				
153	Monomers	*cis*-Isorhapontigenin	C15H14O4	258.270	257	241, 213, 185, 224	318, 220	MS	✓				
154	Monomers	*trans*-Rhaponticin	C21H24O9	420.414	419	**257**, **241**, 281, 299, 323, 405, 389, 243, 169, 395	324, 220	MS	✓				
155	Monomers	*trans*-Pinostilbene	C15H14O3	242.270	241	181, 225, 197, 169	NA	MS	✓				
156	Monomers	*cis*-Pinostilbene	C15H14O3	242.270	241	181, 225, 197, 169	NA	MS	✓				
111	Dimers	Leachianol G	C28H24O7	472.496	471	**387**, 377, 349, 255, 121	280, 218	MS, NMR		✓	✓	✓	
112	Dimers	Leachianol F	C28H24O7	472.496	471	**349**, 453, 255, 287, 153, 241, 121	280, 218	MS, NMR		✓	✓	✓	
114	Dimers	Restrytisol A	C28H24O7	472.486	471	**377**, **255**, 349, 121, 471	280, 221	MS	✓	✓	✓		
115	Dimers	Ampelopsin A	C28H22O7	470.479	469	**345**, 451, 375, 363, 257, 357, 423, 317, 241	283	MS, NMR		✓	✓	✓	✓
116	Dimers	Pallidol	C28H22O6	454.478	453	**359**, 265, 435, 406, 391, 346, 273	284	MS, NMR	✓	✓	✓	✓	✓
118	Dimers	Caraphenol B	C28H22O7	470.473	469	**451**, 281, 363, 375, 227, 423	326, 291	MS		✓	✓		
130	Dimers	Ampelopsin D	C28H22O6	454.478	453	**359**, 361, 437, 215, 343, 199, 255, 289	314, 280	MS, NMR	✓	✓	✓	✓	
131	Dimers	Quadrangularin A	C28H22O6	454.471	453	**359**, 289, 411, 435, 347, 253	314	MS, NMR	✓	✓			
132	Dimers	(+)-*cis*-ε-Viniferin	C28H22O6	454.471	453	435, **347**, 411, 333, 359, 369, 253	286, 201, 230	MS, NMR	✓			✓	
134	Dimers	(+)-*trans*-ε-Viniferin	C28H22O6	454.471	453	**359**, **435**, **347**, 369, 411, 333, 253, 225	327, 285, 308	MS, NMR, DAD	✓	✓	✓	✓	✓
135	Dimers	Viniferifuran	C28H20O6	452.455	451	NA	317, 289, 209	MS, NMR		✓			
136	Dimers	Diptoindonesin A	C34H32O11	616.610	615	**453**, 359, 411, 347, 585	326, 226	MS, NMR		✓		✓	
141	Dimers	*trans*-ω-Viniferin	C28H22O6	454.478	453	**435**, 359, 347, 411, 395, 333, 285	324, 280	MS, NMR	✓		✓	✓	✓
144	Dimers	*cis*-ω-Viniferin	C28H22O6	454.478	453	**435**, 411, 395, 333, 285, 359, 225	294	MS. NMR	✓				
149	Dimers	*trans*-δ-Viniferin	C28H22O6	454.478	453	**435**, **369**, **411**, 347, 333, 359, 225, 409	312, 225	MS, DAD	✓	✓	✓		✓
152	Dimers	*cis*-δ-Viniferin	C28H22O6	454.478	453	**435**, **411**, **369**, 359, 333, 347, 317, 307, 251, 267	285, 232	MS	✓				
157	Dimers	*trans*-ε-Viniferin derivative (dimethylated)	C30H26O6	482.523	481	387, 375, 226, 197, 466	325	MS	✓				
158	Dimers	*trans*-δ-Viniferin derivative (dimethylated)	C30H26O6	482.523	481	397, 361, 439, 387, 463	313	MS	✓				
159	Dimers	*trans*-Scirpusin A	C28H22O7	470.470	469	**375**, 451, 385, 359, 241, 427, 728, 445, 287, 514, 955	320, 286, 204	MS, NMR		✓			
162	Dimers	Maackin A	C28H22O8	486.470	485	244, 226, 137	327, 288, 204	MS, NMR		✓			
164	Dimers	*trans*-ε-Viniferin derivative (γ-lactam ring)	C32H26O7N	936.550	536	NA	NA	MS, NMR			✓		
165	Dimers	*trans*-Resveratrol derivative (γ-lactam ring)	C18H16O4N	310.324	310	NA	NA	MS, NMR			✓		
171	Dimers	Malibatol A	C28H20O7	468.454	467	NA	NA	MS, NMR		✓			
172	Dimers	Ampelopsin F	C28H22O6	454.471	453	NA	282, 220	MS, NMR		✓			
176	Dimers	Viniferal	C35H26O8	574.579	573	NA	NA	MS, NMR		✓			
177	Dimers	Vitisinol C	C27H24O5	428.482	427	NA	358, 279	MS			✓		
178	Dimers	Vitisinol E	*C27H24O6*	444.475	444	NA	281, 230, 204	MS, NMR		✓			
179	Dimers	Vitisinol B	C35H26O8	574.579	573	NA	282, 228, 204	MS, NMR					✓
181	Dimers	Viniferether A	C29H26O7	486.509	485	NA	280, 229	MS, NMR					✓
182	Dimers	Viniferether B	C29H26O7	486.513	485	NA	280, 231	MS, NMR					✓
125	Trimers	Ampelopsin B	C28H22O6	454.478	453	**359**, 243, 211, 183, 265	281, 328	MS, NMR	✓		✓	✓	
139	Trimers	*trans*-Miyabenol C	C42H32O9	680.698	679	**661**, 573, 479, 451, 637, 585, 447	322, 279	MS, NMR	✓	✓	✓	✓	✓
140	Trimers	*cis*-Miyabenol C	C42H32O9	680.699	679	**661**, 573, 479, 451, 637, 585, 447	285	MS, NMR	✓	✓			
142	Trimers	Davidiol A	C42H32O9	680.704	679	**585**, 447, 491, 385, 479, 465, 567	284, 219	MS	✓	✓			
143	Trimers	α-Viniferin	C42H30O9	678.682	677	**571**, 583, 437, 449, 463, 501, 331	284, 309	MS, NMR	✓	✓		✓	
161	Trimers	Ampelopsin C	C42H32O9	680.709	679	NA	283	MS, NMR		✓			✓
169	Trimers	Viniferol D	C42H32O9	680.702	679	NA	NA	MS, NMR		✓			✓
173	Trimers	Ampelopsin E	C42H32O9	680.701	679	NA	325, 285	MS, NMR		✓			✓
129	Tetramers	Hopeaphenol	C56H42O12	906.925	905	**811**, 717, 451, 611, 359, 299	283, 226	MS, NMR		✓	✓	✓	✓
133	Tetramers	Isohopeaphenol	C56H42O12	906.925	905	451, 675, 811, 717, 358, 265	284	MS, NMR	✓	✓	✓	✓	✓
137	Tetramers	Ampelopsin H	C56H42O12	906.925	905	**811**, 717, 705, 793	281	MS, NMR	✓	✓		✓	
138	Tetramers	Vaticanol C-like isomer	C56H42O12	906.929	905	811, 717, 793, 705, 611	281	MS, NMR	✓				
147	Tetramers	Vitisin A (r2-viniferin)	C56H42O12	906.920	905	**811**, 887, 717, 693, 545, 451, 359, 265	328, 285	MS, NMR		✓	✓	✓	✓
148	Tetramers	Vitisifuran A	C56H40O12	904.907	903	NA	322, 232	MS, NMR		✓			
150	Tetramers	Vitisin B (r-viniferin)	C56H42O12	906.920	905	**799**, 887, 811, 717, 545, 451, **359**, **317**	321, 286	MS, NMR		✓	✓	✓	✓
151	Tetramers	Vitisifuran B	C56H40O12	904.907	903	NA	324, 228	MS, NMR		✓			
160	Tetramers	Vitisin C	C56H42O12	906.926	905	NA	NA	MS, NMR		✓			
166	Tetramers	Viniferol A	C56H42O12	906.925	905	559, 813, 361, 453, 651, 541, 801, 783	284, 227	MS, NMR		✓			
167	Tetramers	Viniferol B	C56H42O12	906.929	905	559, 813, 361, 453, 651, 541, 801, 783	283, 225	MS, NMR		✓			
168	Tetramers	Viniferol C	C56H42O12	906.929	905	NA	284, 228	MS, NMR		✓			
170	Tetramers	Viniferol E	C56H44O13	924.940	923	NA	284, 231	MS, NMR					✓
174	Tetramers	Wilsonol C	C56H42O12	906.929	905	NA	231	NMR					✓
175	Tetramers	Heyneanol A	C56H42O12	906.929	905	320, 284	322, 237	NMR					✓
180	Tetramers	Stenophyllol C	C56H42O12	906.923	905	NA	285, 330, 223	MS, NMR					✓
163	Hexamers	Viniphenol A	C84H64O18	1361.391	1360	NA	NA	MS, NMR		✓			

^1^ Other reported names are found in Table S2, where compounds are numbered (ID) according to their elution patterns. ^2^ Another stilbene is reported in the literature as vitisinol E (compound 178), but with the formula C_29_H_26_O_7_, MW of 486,51, [M − H] − (m/z) of 485, λmax CH_3_OH of 358, 279, 253. ^3^ NA = not available or not applicable, MW = Molecular Weight. ^4^ The most abundant fragments are highlighted in bold. ^5^ MS = mass spectrometry detection, NMR = nuclear magnetic resonance detection, DAD = diode array or ultraviolet detection. ^6^ In blue with √ are detected compounds; in light red are undetected compounds or unavailable information.

**Table 3:** Levels (mg/kg) of 92 polyphenols (excluding stilbenes) identified in grapevine leaves.

Id	Compound Name ^1^	Minimum Value ^2,3^	Maximum Value	Mean Value ^4^	Standard Deviation	*N* ^5^	References
63	Quercetin-3-*O*-glucuronide	868.63	46,528.55	**10,305.10**	13,363.51	10	[1,2,4,5,19,59,60,66,75,79,81,82,84,85,86]
58	Quercetin-3-*O*-galactoside	21.72	28,831.11	**7436.94**	9880.90	14	[2,3,5,20,22,73,75,79,81,85]
60	Quercetin-3-*O*-glucoside	27.65	22,610.13	**7256.42**	7628.71	24	[1,2,3,5,19,20,22,59,60,66,73,75,79,80,81,82,84,85,86]
24	Caftaric acid	12.46	14,052.62	**4151.97**	3984.79	18	[3,4,5,19,20,22,59,60,66,73,75,79,81,82,84,85,86]
69	Quercetin-3-*O*-rhamnoside	1210.53	4206.67	**2708.60**	1498.07	2	[1,2]
79	Kaempferol-3-*O*-glucoside	2.56	6203.85	**1730.09**	1812.15	20	[2,3,19,20,22,59,60,66,73,75,79,80,81,84,85,86]
73	Kaempferol-3-*O*-glucuronide	47.92	1698.41	**662.34**	736.83	3	[2,19,66,79,81,82,85,86]
27	Coutaric acid	4.54	1491.02	**635.81**	432.70	10	[3,19,20,22,73,75,79,81,82,85]
53	Myricetin-3-*O*-glucoside	ND	850.12	**291.23**	254.65	8	[2,3,20,22,73,85]
54	Quercetin-3-*O*-rutinoside	1.30	1650.01	**257.51**	473.26	12	[2,4,5,32,73,75,79,81,82,85,87]
71	Kaempferol-3-*O*-rutinoside	0.12	730.01	**200.34**	307.13	4	[2,19,75,79,81,85,86]
32	Fertaric acid	85.48	85.48	**85.48**	0.00	1	[81,82]
7	*p*-Hydroxybenzoic acid	15.80	151.00	**83.40**	67.60	2	[19,32]
42	Gallocatechin gallate	20.10	78.70	**49.40**	29.30	2	[32]
68	Myricetin	1.00	193.28	**44.75**	74.37	5	[1,3,22,81,88]
3	Gallocatechin	4.84	102.00	**40.20**	43.85	3	[32,81,82]
5	Aesculin	1.60	50.70	**25.63**	20.06	3	[32,81]
15	Epigallocatechin	1.67	66.30	**23.77**	30.08	3	[32,81,82]
37	Epigallocatechin gallate	0.04	43.81	**10.69**	16.71	6	[32,73,81,82]
23	Catechin	0.02	76.58	**10.62**	21.44	18	[19,32,59,60,73,75,79,81,82,84,87,88]
46	Ferulic acid	0.008	89.80	**9.07**	25.61	11	[32,59,60,73,79,81,84]
82	Quercetin	0.13	52.17	**8.84**	16.55	16	[1,4,19,32,59,60,73,84,87,88]
6	Procyanidin B1	0.39	25.56	**6.80**	10.83	4	[73,75,79,81,82]
4	Protocatechuic acid	1.25	10.50	**5.88**	4.63	2	[32]
8	Gentisic acid	0.59	8.85	**4.72**	4.13	2	[32]
11	Phlorizin	2.95	2.95	**2.95**	0.00	1	[81,82]
2	Gallic acid	0.01	7.80	**2.77**	2.95	9	[32,79,81,82,87,88]
78	Quercetin-3-(3-*O*-rhamnosyl)glucoside-7-O-rhamnoside	1.32	4.21	**2.77**	1.44	2	[1]
40	Epicatechin	0.01	15.02	**2.46**	4.69	18	[32,59,60,73,75,79,81,82,84,87,88]
94	Quercetin-3-(6-*O*-rhamnosyl)galactoside	0.02	4.02	**2.02**	2.00	2	[1]
29	Procyanidin B2	0.35	5.69	**1.91**	2.21	4	[75,79,81,82]
47	Epicatechin gallate	0.01	8.45	**1.74**	2.57	9	[59,60,81,82,84,88]
28	Chlorogenic acid	0.01	11.50	**1.74**	3.70	8	[32,73,79,88]
35	Caffeic acid	0.003	19.60	**1.68**	4.84	15	[1,32,59,73,79,81,82,84,87,88]
25	Procyanidin B3	0.74	2.41	**1.57**	0.84	2	[75,81,82]
26	Procyanidin B4	0.61	2.38	**1.49**	0.89	2	[75,81,86]
93	Isorhamnetin-3-*O*-glucoside	1.48	1.48	**1.48**	0.00	1	[2,80,81,85]
84	Kaempferol	0.01	6.77	**1.28**	2.19	8	[1,32,59,60,84,86,88]
34	Cyanidin-3-*O*-glucoside	0.01	6.40	**1.17**	2.04	8	[4,5,59,60,77,78,79,84,88]
98	Isorhamnetin-3-*O*-rutinoside	1.12	1.12	**1.12**	0.00	1	[2,81]
43	*p*-Coumaric acid	0.01	8.17	**0.92**	2.42	10	[32,59,60,79,84,88]
64	Apigenin-7-*O*-glucoside	0.09	1.60	**0.85**	0.75	2	[1]
10	Procyanidin A1	0.72	0.72	**0.72**	0.00	1	[75]
66	Luteolin-7-*O*-glucoside	0.02	1.91	**0.60**	0.69	5	[1,4,73,81,82]
48	Sinapic acid	0.55	0.55	**0.55**	0.00	1	[81]
50	Ellagic acid	0.06	0.77	**0.41**	0.36	2	[32]
41	Taxifolin	0.37	0.37	**0.37**	0.00	1	[81]
83	Cinnamic acid	0.17	0.51	**0.34**	0.17	2	[79]
75	Quercetin-3-(3-*O*-arabinosyl)glucoside	0.31	0.31	**0.31**	0.00	1	[2,81]
89	Kaempferol-3-*O*-xyloside	0.23	0.23	**0.23**	0.00	1	[2,75]
45	Peonidin-3-*O*-glucoside	0.01	0.60	**0.20**	0.24	4	[5,77,78,79,88]
30	Vanillic acid	0.01	0.54	**0.19**	0.19	5	[77,78,79,81,88]
14	1-*O*-Sinapoyl-*β*-D-glucose	0.15	0.15	**0.15**	0.00	1	[75]
70	Kaempferol-3-*O*-galactoside	0.06	0.06	**0.06**	0.00	1	[2,75,85,86]
36	Syringic acid	0.01	0.07	**0.04**	0.03	2	[88]
52	Malvidin-3-*O*-glucoside	0.01	0.06	**0.04**	0.03	2	[1,2,3,4,5,19,32,59,66,75,77,78,79,80,81,82,84,85,86,88]
16	1-*O*-(4-Coumaroyl)-glucose	0.03	0.03	**0.03**	0.00	1	[75]
1	Quinic acid	NQ	NQ	**NQ**	NQ	0	[4,19]
9	γ-Resorcylic acid	NQ	NQ	**NQ**	NQ	0	[82]
12	Fraxin	NQ	NQ	**NQ**	NQ	0	[82]
13	Aesculetin	NQ	NQ	**NQ**	NQ	0	[19]
17	Umbelliferone	NQ	NQ	**NQ**	NQ	0	[19]
20	1-Caffeoyl-*β*-D-glucose	NQ	NQ	**NQ**	NQ	0	[81]
21	Ferulic acid pentose	NQ	NQ	**NQ**	NQ	0	[19]
22	Caftaric acid isomer	NQ	NQ	**NQ**	NQ	0	[85]
33	Delphinidin-3-*O*-glucoside	NQ	NQ	**NQ**	NQ	0	[77,78,79]
44	Petunidin-3-*O*-glucoside	NQ	NQ	**NQ**	NQ	0	[77,78,79]
49	Myricetin-3-*O*-galactoside	NQ	NQ	**NQ**	NQ	0	[2,79,85]
51	Myricetin-3-*O*-glucuronide	NQ	NQ	**NQ**	NQ	0	[2,79]
59	Petunidin-3-(6-*O*-acetyl)glucoside	NQ	NQ	**NQ**	NQ	0	[77]
61	Catechin gallate	NQ	NQ	**NQ**	NQ	0	[86]
62	Peonidin-3-(6-*O*-acetyl)glucoside	NQ	NQ	**NQ**	NQ	0	[79]
65	Myricetin-3-*O*-rhamnoside	NQ	NQ	**NQ**	NQ	0	[2]
67	Malvidin-3-(6-*O*-acetyl)glucoside	NQ	NQ	**NQ**	NQ	0	[77,79]
72	Cyanidin-3-(6-*O*-coumaroyl)glucoside	NQ	NQ	**NQ**	NQ	0	[77]
74	Quercetin-3-(6-*O*-acetyl)glucoside	NQ	NQ	**NQ**	NQ	0	[19]
76	Quercetin-3-(7-O-glucosyl)glucuronide	NQ	NQ	**NQ**	NQ	0	[80]
80	Petunidin-3-(6-*O*-coumaroyl)glucoside	NQ	NQ	**NQ**	NQ	0	[77]
81	Peonidin-3-(6-*O*-coumaroyl)glucoside	NQ	NQ	**NQ**	NQ	0	[2,77,79]
85	Malvidin-3-(6-*O*-coumaroyl)glucoside	NQ	NQ	**NQ**	NQ	0	[77,79]
88	Hesperetin	NQ	NQ	**NQ**	NQ	0	[4]
90	Kaempferol-3-*O*-rhamnoside	NQ	NQ	**NQ**	NQ	0	[2]
92	Isorhamnetin-3-*O*-galactoside	NQ	NQ	**NQ**	NQ	0	[2,85]
95	Eriodictyol-7-*O*-glucoside	NQ	NQ	**NQ**	NQ	0	[19]
96	Isorhamnetin-3-*O*-arabinose	NQ	NQ	**NQ**	NQ	0	[2]
97	Isorhamnetin-3-*O*-glucuronide	NQ	NQ	**NQ**	NQ	0	[2]
100	Isorhamnetin-3(4-*O*-rhamnosyl)rutinoside	NQ	NQ	**NQ**	NQ	0	[2,80,85]
101	Kaempferol-3-(6-*O*-coumaroyl)glucoside	NQ	NQ	**NQ**	NQ	0	[4]
102	Kaempferol-3 (7-O-glucosyl)galactoside	NQ	NQ	**NQ**	NQ	0	[80]
103	Naringenin	NQ	NQ	**NQ**	NQ	0	[82]
104	Naringenin-7-*O*-glucoside	NQ	NQ	**NQ**	NQ	0	[82]
105	Diquercetin-3-(3-*O*-glucosyl)glucuronide	NQ	NQ	**NQ**	NQ	0	[66]

^1^ Compounds 33, 43, 52, 59, 62, 67, 72, 76, 80, 83, 85, and 102 are detected using only UV. ^2^ ND = not detected. ^3^ NQ = not quantified by the authors. ^4^ Fresh and dry weight data were combined for the calculations, without any conversion. ^5^ *N* = number of data points used in the calculation of the mean value, and made of minimum, maximum, and average values extracted from each reference.

**Table 4:** Levels (mg/kg) of 40 stilbenic compounds identified in grapevine leaves.

Id	Compound Name	Minimum Value ^1,2^	Maximum Value	Mean Value ^3^	Standard Deviation	*N* ^4^	References
123	*trans*-Resveratrol	ND	1886.80	**137.88**	444.15	24	[6,10,11,19,23,32,59,60,61,62,63,64,68,70,73,81,82,84,87,88,89]
138	Vaticanol C-like isomer	ND	226.80	**96.85**	102.35	6	[61,81,82,89]
121	*cis*-Piceid	ND	368.40	**78.38**	132.57	6	[10,62,63,64,68,81,82]
119	*trans*-Piceatannol	ND	232.10	**78.04**	108.94	5	[23,63,82,88]
137	Ampelopsin H	ND	226.80	**76.08**	106.58	6	[61,81,82,89]
143	α-Viniferin	ND	189.06	**71.61**	75.19	6	[10,61,81,89]
140	*cis*-Miyabenol C	ND	148.60	**50.67**	69.29	6	[61,81,82,89]
110	*cis*-Resveratrol-3-*O*-glucoside	ND	232.63	**47.41**	83.30	6	[59,60,84]
113	*trans*-Piceid	ND	170.23	**44.71**	64.69	17	[6,10,11,23,61,62,63,64,68,70,73,81,82,89]
139	*trans*-Miyabenol C	ND	121.30	**41.57**	56.43	6	[61,81,82,89]
149	*trans*-δ-Viniferin	1.09	165.71	**35.55**	53.31	8	[6,10,11,62,68,70,82]
120	*cis*-Resveratroloside	15.20	37.50	**26.35**	11.15	2	[63]
134	(+)-*trans*-ε-Viniferin	ND	98.20	**25.11**	35.79	15	[6,10,11,23,61,62,68,70,73,81,82,89]
130	Ampelopsin D	ND	67.60	**22.78**	31.70	6	[10,61,81,82,89]
141	*trans*-ω-Viniferin	ND	63.55	**21.35**	29.84	6	[10,61,81,82,89]
144	*cis*-ω-Viniferin	ND	63.55	**21.24**	29.92	6	[10,61,81,82,89]
128	*cis*-Resveratrol	ND	53.10	**19.46**	20.50	4	[62,63,64,73,82]
107	*trans*-Resveratroloside	7.50	21.80	**14.65**	7.15	2	[63]
116	Pallidol	ND	26.71	**11.52**	12.09	6	[61,81,82,89]
131	Quadrangularin A	ND	33.80	**11.29**	15.92	6	[10,61,81,82,89]
133	Isohopeaphenol	ND	131.17	**7.12**	12.33	6	[61,81,82,89]
153	*cis*-Isorhapontigenin	0.10	13.00	**6.55**	6.45	2	[63]
122	*trans*-Isorhapontin	0.07	21.30	**6.44**	8.69	4	[63,81,82]
126	*trans*-Isorhapontigenin	0.10	9.60	**4.85**	4.75	2	[63]
145	*trans*-Pterostilbene	ND	10.83	**3.92**	4.24	10	[6,10,61,62,64,68,70,82,89]
132	(+)-*cis*-ε-Viniferin	ND	7.31	**1.83**	3.17	4	[10,62,68,82,89]
106	*trans*-Astringin	0.04	7.60	**3.02**	3.09	4	[63,81,82]
152	*cis*-δ-Viniferin	ND	3.42	**1.71**	1.71	2	[62,68]
127	*trans*-Pinostilbene-4′-*O*-glucoside	0.10	3.30	**1.70**	1.60	2	[63]
117	*cis*-Astringin	0.20	2.10	**1.15**	0.95	2	[63]
155	*trans*-Pinostilbene	0.10	2.00	**1.05**	0.95	2	[63]
154	*trans*-Rhaponticin	0.10	1.80	**0.95**	0.85	2	[63]
156	*cis*-Pinostilbene	0.10	0.30	**0.20**	0.10	2	[63]
114	Restrytisol A	NQ	NQ	**NQ**	NQ	0	[10]
124	2,4,6-Trihydroxyphenanthrene-2-*O*-glucoside	NQ	NQ	**NQ**	NQ	0	[64]
125	Ampelopsin B	NQ	NQ	**NQ**	NQ	0	[10]
142	Davidiol A	NQ	NQ	**NQ**	NQ	0	[10]
146	*cis*-Pterostilbene	NQ	NQ	**NQ**	NQ	0	[10]
157	*trans*-ε-Viniferin derivative (dimethylated)	NQ	NQ	**NQ**	NQ	0	[10]
158	*trans*-δ-Viniferin derivative (dimethylated)	NQ	NQ	**NQ**	NQ	0	[10]

^1^ ND = not detected. ^2^ NQ = not quantified by the authors. ^3^ Fresh and dry weight data were combined for the calculations, without any conversion. ^4^ *N* = number of data points used in the calculation of the mean value, and made of minimum, maximum, and average values extracted from each reference.

**Table 5:** Levels (mg/kg) of 41 polyphenols (excluding stilbenes) identified in grapevine stems.

Id	Compound Name ^1^	Minimum Value ^2^	Maximum Value	Mean Value ^3^	Standard Deviation	*N* ^4^	References
58	Quercetin-3-*O*-galactoside	1920.34	41,831.70	**17,403.61**	15,457.56	4	[21,50]
23	Catechin	283.72	98,290.95	**14,900.45**	27,191.10	12	[9,21,37,50,53,55,69,74,90]
2	Gallic acid	386.54	32,960.41	**10,307.36**	13,374.20	4	[9,21,34,37,55]
40	Epicatechin	193.61	33,154.03	**9251.64**	12,435.12	14	[9,21,33,50,53,54,55,69,90]
6	Procyanidin B1	215.36	50,709.00	**9216.18**	14,385.61	10	[9,33,37,50,53,54,55,69]
19	Procyanidin T2	1388.90	35,015.04	**9100.99**	8406.54	2	[9,34,50,55,69]
25	Procyanidin B3	186.04	23,108.65	**8724.23**	5791.29	4	[9,21,55,69]
47	Epicatechin gallate	2371.55	9862.08	**6362.96**	2950.30	6	[9,21,33,54,55]
18	Procyanidin C1	305.51	9710.00	**5007.76**	4702.25	2	[9,50,55,69]
82	Quercetin	321.88	8210.20	**4266.04**	3944.16	2	[21]
24	Caftaric acid	110.35	16,110.62	**3373.18**	5723.49	6	[33,54,74,87]
26	Procyanidin B4	131.00	4355.20	**2243.10**	2112.10	2	[69]
39	Procyanidin dimer gallate	110.04	4358.12	**2234.08**	2124.04	2	[9,33,34,50,54,69]
29	Procyanidin B2	10.49	6670.76	**2056.93**	2735.52	4	[9,21,50,53,55,69,74]
60	Quercetin-3-*O*-glucoside	29.88	7270.12	**1785.38**	2544.06	6	[9,21,37,53,55,90]
69	Quercetin-3-*O*-rhamnoside	320.20	2820.00	**1570.10**	1249.90	2	[21]
10	Procyanidin A1	674.91	1833.85	**1254.38**	579.47	4	[33,50,54]
84	Kaempferol	70.12	1830.57	**950.35**	880.23	2	[21]
63	Quercetin-3-*O*-glucuronide	391.52	1424.35	**908.82**	469.54	6	[33,34,37,54,74]
87	Malvidin-3-*O*-rutinoside	451.00	628.77	**539.89**	88.88	4	[33,54]
52	Malvidin-3-*O*-glucoside	224.88	801.37	**513.13**	288.25	4	[33,54]
43	*p*-Coumaric acid	12.00	934.08	**473.04**	461.04	2	[9,21,55]
35	Caffeic acid	10.18	647.32	**328.75**	318.57	2	[9,21,55]
38	Prodelphinidin A-type	27.46	292.88	**160.17**	132.71	2	[33,50,54]
99	Isorhamnetin-3-(6-*O*-feruloyl)glucoside	81.10	115.07	**98.09**	16.99	4	[33,54]
86	Malvidin-3-(6-*O*-caffeoyl)glucoside	47.33	119.20	**83.27**	35.94	4	[33,54]
71	Kaempferol-3-*O*-rutinoside	21.99	127.39	**74.69**	52.70	4	[33,54]
54	Quercetin-3-*O*-rutinoside	10.55	126.73	**65.84**	46.33	6	[21,33,34,54,74,90]
79	Kaempferol-3-*O*-glucoside	20.14	79.08	**49.61**	29.47	4	[33,54]
36	Syringic acid	6.48	32.23	**19.36**	12.88	2	[21]
46	Ferulic acid	8.01	25.55	**16.78**	8.77	2	[9,21,55]
16	1-*O*-(4-Coumaroyl)-glucose	NQ	NQ	**NQ**	NQ	0	[53]
20	1-Caffeoyl-*β*-D-glucose	NQ	NQ	**NQ**	NQ	0	[53]
21	Ferulic acid pentose	NQ	NQ	**NQ**	NQ	0	[9,55]
31	Chicoric acid	NQ	NQ	**NQ**	NQ	0	[9,55]
55	Taxifolin-*O*-pentoside	NQ	NQ	**NQ**	NQ	0	[9,55]
56	Taxifolin-3-*O*-glucoside	NQ	NQ	**NQ**	NQ	0	[53]
57	Taxifolin-3-*O*-rhamnoside	NQ	NQ	**NQ**	NQ	0	[9,34,55,74]
61	Catechin gallate	NQ	NQ	**NQ**	NQ	0	[53]
77	Quercetin-3-*O*-arabinose	NQ	NQ	**NQ**	NQ	0	[50]
91	Dihydrokaempferol-3-*O*-rhamnoside	NQ	NQ	**NQ**	NQ	0	[9,55]

^1^ Compound 52 is detected using only UV. ^2^ NQ = not quantified by the authors. ^3^ Fresh and dry weight data were combined for the calculations, without any conversion. ^4^ *N* = number of data point used in the calculation of the mean value, and made of minimum, maximum, and average values extracted from each reference.

**Table 6:** Levels (mg/kg) of 47 stilbenic compounds identified in grapevine stems.

Id	Compound Name	Minimum Value ^1,2^	Maximum Value	Mean Value ^3^	Standard Deviation	*N* ^4^	References
123	*trans*-Resveratrol	ND	2130.00	**506.41**	570.04	6	[9,23,26,27,53,55,58,91]
134	(+)-*trans*-ε-Viniferin	14.30	1400.67	**433.49**	765.23	6	[9,23,26,27,28,53,55,56,58,67,91]
130	Ampelopsin D	ND	130.00	**65.00**	65.00	2	[53]
150	Vitisin B (r-Viniferin)	6.80	61.10	**33.95**	27.15	2	[26,27,28,53,58,67,91]
113	*trans*-Piceid	14.52	14.52	**14.52**	0.00	1	[9,23,26,27,53,55]
126	*trans*-Isorhapontigenin	ND	19.80	**9.90**	9.90	2	[91]
119	*trans*-Piceatannol	ND	21.10	**7.42**	9.68	3	[9,23,26,27,53,55,58,91]
149	*trans*-δ-Viniferin	4.86	4.86	**4.86**	0.00	1	[23]
106	*trans*-Astringin	NQ	NQ	**NQ**	NQ	0	[9,53,55]
107	*trans*-Resveratroloside	NQ	NQ	**NQ**	NQ	0	[53]
108	*trans*-Resveratrol-2-*C*-glucoside	NQ	NQ	**NQ**	NQ	0	[53]
109	*trans*-Resveratrol-10-*C*-glucoside	NQ	NQ	**NQ**	NQ	0	[56]
110	*trans*-Resveratrol-*O*-glucoside	NQ	NQ	**NQ**	NQ	0	[56]
111	Leachianol G	NQ	NQ	**NQ**	NQ	0	[26,27]
112	Leachianol F	NQ	NQ	**NQ**	NQ	0	[26,27]
114	Restrytisol A	NQ	NQ	**NQ**	NQ	0	[58]
115	Ampelopsin A	NQ	NQ	**NQ**	NQ	0	[26,27,28,53,56,58]
116	Pallidol	NQ	NQ	**NQ**	NQ	0	[9,26,27,55]
118	Caraphenol B	NQ	NQ	**NQ**	NQ	0	[53]
129	Hopeaphenol	NQ	NQ	**NQ**	NQ	0	[9,26,53,55,56,58]
131	Quadrangularin A	NQ	NQ	**NQ**	NQ	0	[26,27,53]
133	Isohopeaphenol	NQ	NQ	**NQ**	NQ	0	[26,27,53,56,67]
135	Viniferifuran	NQ	NQ	**NQ**	NQ	0	[67]
136	Diptoindonesin A	NQ	NQ	**NQ**	NQ	0	[26,27,53]
137	Ampelopsin H	NQ	NQ	**NQ**	NQ	0	[26,27]
139	*trans*-Miyabenol C	NQ	NQ	**NQ**	NQ	0	[26,27,53,58]
140	*cis*-Miyabenol C	NQ	NQ	**NQ**	NQ	0	[53]
142	Davidiol A	NQ	NQ	**NQ**	NQ	0	[26,27,53]
143	α-Viniferin	NQ	NQ	**NQ**	NQ	0	[9,55]
145	*trans*-Pterostilbene	NQ	NQ	**NQ**	NQ	0	[9,26,27,55]
147	Vitisin A (r2-Viniferin)	NQ	NQ	**NQ**	NQ	0	[28,56,58,67]
148	Vitisifuran A	NQ	NQ	**NQ**	NQ	0	[67]
151	Vitisifuran B	NQ	NQ	**NQ**	NQ	0	[67]
159	*trans*-Scirpusin A	NQ	NQ	**NQ**	NQ	0	[26,27,53]
160	Vitisin C	NQ	NQ	**NQ**	NQ	0	[26,27,67]
161	Ampelopsin C	NQ	NQ	**NQ**	NQ	0	[26,27]
162	Maackin A	NQ	NQ	**NQ**	NQ	0	[26,27]
163	Viniphenol A	NQ	NQ	**NQ**	NQ	0	[26,27]
166	Viniferol A	NQ	NQ	**NQ**	NQ	0	[56]
167	Viniferol B	NQ	NQ	**NQ**	NQ	0	[56]
168	Viniferol C	NQ	NQ	**NQ**	NQ	0	[56]
169	Viniferol D	NQ	NQ	**NQ**	NQ	0	[67]
171	Malibatol A	NQ	NQ	**NQ**	NQ	0	[56]
172	Ampelopsin F	NQ	NQ	**NQ**	NQ	0	[56]
173	Ampelopsin E	NQ	NQ	**NQ**	NQ	0	[56]
176	Viniferal	NQ	NQ	**NQ**	NQ	0	[67]
178	Vitisinol E	NQ	NQ	**NQ**	NQ	0	[28]

^1^ ND = not detected. ^2^ NQ = not quantified by the authors. ^3^ Fresh and dry weight data were combined for the calculations, without any conversion. ^4^ *N* = number of data points used in the calculation of the mean value, and made of minimum, maximum, and average values extracted from each reference.

**Table 7:** Levels (mg/kg) of 26 stilbenic compounds identified in grapevine canes.

Id	Compound Name	Minimum Value ^1,2^	Maximum Value	Mean Value ^3^	Standard Deviation	*N* ^4^	References
123	*trans*-Resveratrol	ND	6526.29	**2797.17**	2559.72	17	[7,12,24,25,40,48,49,50,51,52,58,72]
134	(+)-*trans*-ε-Viniferin	21.00	12,612.22	**2449.25**	3197.26	13	[7,12,24,25,40,50,51,52,58]
133	Isohopeaphenol	ND	3521.52	**851.43**	1133.46	7	[12,24,51,52]
150	Vitisin B (r-Viniferin)	0.01	2159.00	**668.08**	818.46	10	[7,12,24,40,50,51,52,58]
119	*trans*-Piceatannol	0.50	1710.24	**583.88**	799.71	11	[7,12,24,25,40,50,51,52,58]
141	*trans*-ω-Viniferin	ND	1714.63	**556.41**	628.60	3	[7,12,24,25,50]
129	Hopeaphenol	ND	1439.21	**511.39**	585.40	9	[12,24,25,40,50,51,52,58]
116	Pallidol	4.00	1276.43	**440.81**	591.08	3	[12,24,40,50]
139	*trans*-Miyabenol C	0.01	2108.47	**412.53**	702.07	7	[7,12,24,25,40,51,52,58]
115	Ampelopsin A	0.01	1684.16	**370.88**	534.32	8	[12,24,25,40,50,51,52,58]
147	Vitisin A (r2-Viniferin)	43.00	717.55	**293.18**	301.67	3	[12,24,25,50,51,58]
149	*trans*-δ-Viniferin	9.00	43.00	**26.00**	17.00	2	[24,50]
113	*trans*-Piceid	0.50	36.21	**13.50**	16.11	5	[7,40,48,49,50]
177	Vitisinol C	1.00	29.00	**15.00**	14.00	2	[24]
108	*trans*-Resveratrol-2-*C*-glucoside	NQ	NQ	**NQ**	NQ	0	[7]
110	*trans*-Resveratrol-*O*-glucoside	NQ	NQ	**NQ**	NQ	0	[72]
111	Leachianol G	NQ	NQ	**NQ**	NQ	0	[50]
112	Leachianol F	NQ	NQ	**NQ**	NQ	0	[50]
114	Restrytisol A	NQ	NQ	**NQ**	NQ	0	[50,58]
118	Caraphenol B	NQ	NQ	**NQ**	NQ	0	[72]
121	*cis*-Piceid	NQ	NQ	**NQ**	NQ	0	[50]
125	Ampelopsin B	NQ	NQ	**NQ**	NQ	0	[40]
126	*trans*-Isorhapontigenin	NQ	NQ	**NQ**	NQ	0	[50]
130	Ampelopsin D	NQ	NQ	**NQ**	NQ	0	[50]
164	*trans*-ε-Viniferin derivative (γ-lactam ring)	NQ	NQ	**NQ**	NQ	0	[25]
165	*trans*-Resveratrol derivative (γ-lactam ring)	NQ	NQ	**NQ**	NQ	0	[25]

^1^ ND = not detected. ^2^ NQ = not quantified by the authors. ^3^ Fresh and dry weight data were combined for the calculations, without any conversion. ^4^ *N* = number of data points used in the calculation of the mean value, and made of minimum, maximum, and average values extracted from each reference.

**Table 8:** Levels (mg/kg) of 23 polyphenols (excluding stilbenes) identified in grapevine canes.

Id	Compound Name	Minimum Value ^1^	Maximum Value	Mean Value ^2^	Standard Deviation	*N* ^3^	References
23	Catechin	65.16	6735.24	**1747.01**	2525.88	10	[46,48,49,50,83]
48	Sinapic acid	26.41	2283.20	**1154.81**	1128.40	4	[46,47,83]
6	Procyanidin B1	215.36	806.87	**511.12**	295.76	2	[50]
40	Epicatechin	45.53	896.17	**269.40**	289.87	6	[48,49,50]
46	Ferulic acid	0.92	650.13	**165.63**	279.74	8	[46,47,48,49,83]
2	Gallic acid	7.21	570.13	**165.06**	234.37	8	[46,47,48,49,83]
38	Prodelphinidin A-type	27.46	292.88	**160.17**	132.71	2	[50]
4	Protocatechuic acid	3.25	379.85	**103.31**	159.84	8	[46,47,48,49,83]
24	Caftaric acid	18.64	77.60	**48.12**	29.48	4	[48,49]
7	*p*-Hydroxybenzoic acid	0.01	95.22	**47.62**	47.61	4	[46,47,83]
30	Vanillic acid	0.01	152.10	**40.13**	64.74	8	[46,47,48,49,83]
36	Syringic acid	0.01	113.09	**31.05**	47.43	8	[46,47,48,49,83]
50	Ellagic acid	0.01	53.25	**18.78**	20.99	8	[46,48,49,83]
27	Coutaric acid	5.20	19.39	**12.30**	7.10	4	[48,49]
43	*p*-Coumaric acid	0.01	31.20	**11.13**	11.97	8	[46,47,48,49,83]
35	Caffeic acid	1.15	3.43	**2.29**	1.14	4	[47,48,49]
10	Procyanidin A1	NQ	NQ	**NQ**	NQ	0	[50]
18	Procyanidin C1	NQ	NQ	**NQ**	NQ	0	[50]
19	Procyanidin T2	NQ	NQ	**NQ**	NQ	0	[50]
29	Procyanidin B2	NQ	NQ	**NQ**	NQ	0	[50]
39	Procyanidin dimer gallate	NQ	NQ	**NQ**	NQ	0	[50]
58	Quercetin-3-*O*-galactoside	NQ	NQ	**NQ**	NQ	0	[50]
77	Quercetin-3-*O*-arabinose	NQ	NQ	**NQ**	NQ	0	[50]

^1^ NQ = not quantified by the authors. ^2^ Fresh and dry weight data were combined for the calculations, without any conversion. ^3^ *N* = number of data points used in the calculation of the mean value, and made of minimum, maximum, and average values extracted from each reference.

**Table 9:** Levels (mg/kg) of 23 stilbenic compounds identified in grapevine woods.

Id	Compound Name	Minimum Value ^1,2^	Maximum Value	Mean Value ^3^	Standard Deviation	*N* ^4^	References
134	(+)-*trans*-ε-Viniferin	122.57	14,080.88	**8263.87**	4866.89	7	[12,13,43,45]
132	(+)-*cis*-ε-Viniferin	2504.08	14,023.65	**3851.97**	5759.79	2	[13]
133	Isohopeaphenol	11.68	7913.00	**2429.69**	2544.59	7	[12,13,43,45]
143	α-Viniferin	ND	4732.06	**2366.03**	2366.03	2	[13]
123	*trans*-Resveratrol	15.11	3604.04	**2195.12**	1414.54	7	[12,13,43,45]
137	Ampelopsin H	1144.77	2518.08	**1831.43**	686.66	2	[13]
116	Pallidol	410.60	2602.15	**1647.36**	916.64	3	[12,13]
111	Leachianol G	350.32	2800.34	**1575.33**	1225.01	2	[13]
129	Hopeaphenol	20.09	5006.77	**1570.13**	1683.09	7	[12,13,43,45]
141	*trans*-ω-Viniferin	1554.16	1554.16	**1554.16**	0.00	1	[12]
112	Leachianol F	35.57	2805.13	**1420.35**	1384.78	2	[13]
115	Ampelopsin A	151.00	3684.01	**1345.17**	1541.97	5	[12,13,45]
147	Vitisin A (r2-Viniferin)	1298.67	1298.67	**1298.67**	0.00	1	[12]
128	*cis*-Resveratrol	780.58	3609.66	**774.64**	1192.86	2	[13]
110	*trans*-Resveratrol-*O*-glucoside	131.00	1090.00	**610.50**	479.50	2	[44]
150	Vitisin B (r-Viniferin)	569.18	569.18	**569.18**	0.00	1	[12]
139	*trans*-Miyabenol C	ND	1339.51	**430.02**	482.17	5	[12,13,45]
125	Ampelopsin B	ND	493.44	**246.72**	246.72	2	[13]
119	*trans*-Piceatannol	38.00	378.07	**160.69**	154.14	3	[12,45]
130	Ampelopsin D	10.51	310.22	**160.37**	149.86	2	[43]
113	*trans*-Piceid	35.00	50.00	**42.50**	7.50	2	[45]
136	Diptoindonesin A	9.78	57.70	**33.74**	23.96	2	[44]
106	*trans*-Astringin	2.56	24.00	**13.28**	10.72	2	[44]

^1^ ND = not detected. ^2^ NQ = not quantified by the authors. ^3^ Fresh and dry weight data were combined for the calculations, without any conversion. ^4^ *N* = number of data points used in the calculation of the mean value, and made of minimum, maximum, and average values extracted from each reference.

**Table 10:** Levels (mg/kg) of 24 stilbenic compounds identified in grapevine roots.

Id	Compound Name	Minimum Value ^1^	Maximum Value	Mean Value ^2^	Standard Deviation	*N* ^3^	References
134	(+)-*trans*-ε-Viniferin	125.10	18,000.98	**6475.95**	8163.57	3	[12,23,30,43]
150	Vitisin B (r-Viniferin)	11.10	12,829.85	**6420.48**	6409.38	2	[12,30,41,42]
129	Hopeaphenol	1814.90	1814.90	**1814.90**	0.00	1	[12,41,42]
115	Ampelopsin A	15.60	2178.23	**1096.92**	1081.32	2	[12,29,30,41,42]
147	Vitisin A (r2-viniferin)	87.10	2093.13	**1090.12**	1003.02	2	[12,30]
133	Isohopeaphenol	529.54	529.54	**529.54**	0.00	1	[12]
123	*trans*-Resveratrol	46.30	1095.24	**503.25**	438.74	3	[12,23,29,30]
141	*trans*-ω-Viniferin	127.70	127.70	**127.70**	0.00	1	[12]
139	*trans*-Miyabenol C	12.70	212.34	**112.52**	99.82	2	[12,30]
113	*trans*-Piceid	112.07	112.07	**112.07**	0.00	1	[23,29]
116	Pallidol	73.06	73.06	**73.06**	0.00	1	[12,29]
119	*trans*-Piceatannol	4.20	121.33	**47.18**	52.66	3	[12,23,30]
149	*trans*-δ-viniferin	32.77	32.77	**32.77**	0.00	1	[23]
121	*cis*-Piceid	NQ	NQ	**NQ**	NQ	0	[29]
161	Ampelopsin C	NQ	NQ	**NQ**	NQ	0	[41,42]
169	Viniferol D	NQ	NQ	**NQ**	NQ	0	[41,42]
170	Viniferol E	NQ	NQ	**NQ**	NQ	0	[41,42]
173	Ampelopsin E	NQ	NQ	**NQ**	NQ	0	[41,42]
174	Wilsonol C	NQ	NQ	**NQ**	NQ	0	[29]
175	Heyneanol A	NQ	NQ	**NQ**	NQ	0	[29]
179	Vitisinol B	NQ	NQ	**NQ**	NQ	0	[41,42]
180	Stenophyllol C	NQ	NQ	**NQ**	NQ	0	[41,42]
181	Viniferether A	NQ	NQ	**NQ**	NQ	0	[41,42]
182	Viniferether B	NQ	NQ	**NQ**	NQ	0	[41,42]

^1^ NQ = not quantified by the authors. ^2^ Fresh and dry weight data were combined for the calculations, without any conversion. ^3^ *N* = number of data points used in the calculation of the mean value, and made of minimum, maximum, and average values extracted from each reference.

## Supplementary Materials

The following are available online at https://www.mdpi.com/2076-3921/9/5/398/s1, Table S1: Reference source for phenolic acids, flavonoids and coumarins identified in the vegetative organs of grapevine (*Vitis* *vinifera* L.). Table S2: Reference source for stilbenes identified in the vegetative organs of grapevine (*Vitis* *vinifera* L.).
